# Mechanisms of Motor-Independent Membrane Remodeling Driven by Dynamic Microtubules

**DOI:** 10.1016/j.cub.2020.01.036

**Published:** 2020-03-23

**Authors:** Ruddi Rodríguez-García, Vladimir A. Volkov, Chiung-Yi Chen, Eugene A. Katrukha, Natacha Olieric, Amol Aher, Ilya Grigoriev, Magdalena Preciado López, Michel O. Steinmetz, Lukas C. Kapitein, Gijsje Koenderink, Marileen Dogterom, Anna Akhmanova

**Affiliations:** 1 Cell Biology, Department of Biology, Faculty of Science, Utrecht University, Padualaan 8, Utrecht 3584, the Netherlands; 2 Department of Bionanoscience, Kavli Institute of Nanoscience, Delft University of Technology, Van der Maasweg 9, Delft 2629, the Netherlands; 3 Laboratory of Biomolecular Research, Division of Biology and Chemistry, Paul Scherrer Institut, Forschungsstrasse 111, Villigen 5232, Switzerland; 4 University of Basel, Biozentrum, Klingelbergstrasse, Basel 4056, Switzerland; 5 Department of Living Matter, AMOLF, Science Park 104, Amsterdam 1098, the Netherlands

**Keywords:** microtubule, membrane, +TIP, EB1, STIM1, kinesin, tip-attachement complex, in vitro reconstitution, optical trap, force generation

## Abstract

Microtubule-dependent organization of membranous organelles occurs through motor-based pulling and by coupling microtubule dynamics to membrane remodeling. For example, tubules of endoplasmic reticulum (ER) can be extended by kinesin- and dynein-mediated transport and through the association with the tips of dynamic microtubules. The binding between ER and growing microtubule plus ends requires End Binding (EB) proteins and the transmembrane protein STIM1, which form a tip-attachment complex (TAC), but it is unknown whether these proteins are sufficient for membrane remodeling. Furthermore, EBs and their partners undergo rapid turnover at microtubule ends, and it is unclear how highly transient protein-protein interactions can induce load-bearing processive motion. Here, we reconstituted membrane tubulation in a minimal system with giant unilamellar vesicles, dynamic microtubules, an EB protein, and a membrane-bound protein that can interact with EBs and microtubules. We showed that these components are sufficient to drive membrane remodeling by three mechanisms: membrane tubulation induced by growing microtubule ends, motor-independent membrane sliding along microtubule shafts, and membrane pulling by shrinking microtubules. Experiments and modeling demonstrated that the first two mechanisms can be explained by adhesion-driven biased membrane spreading on microtubules. Optical trapping revealed that growing and shrinking microtubule ends can exert forces of ∼0.5 and ∼5 pN, respectively, through attached proteins. Rapidly exchanging molecules that connect membranes to dynamic microtubules can thus bear a sufficient load to induce membrane deformation and motility. Furthermore, combining TAC components and a membrane-attached kinesin in the same *in vitro* assays demonstrated that they can cooperate in promoting membrane tubule extension.

## Highlights

• Proteins transiently binding to dynamic microtubule ends can drive cargo motility
• Dynamic microtubules can remodel membranes through tip-attachment complexes (TACs)
• An EB protein and its membrane-bound partner are sufficient to form a TAC
• TACs and motor proteins can cooperate to extend membrane tubes

## Graphical Abstract

**Figure:**
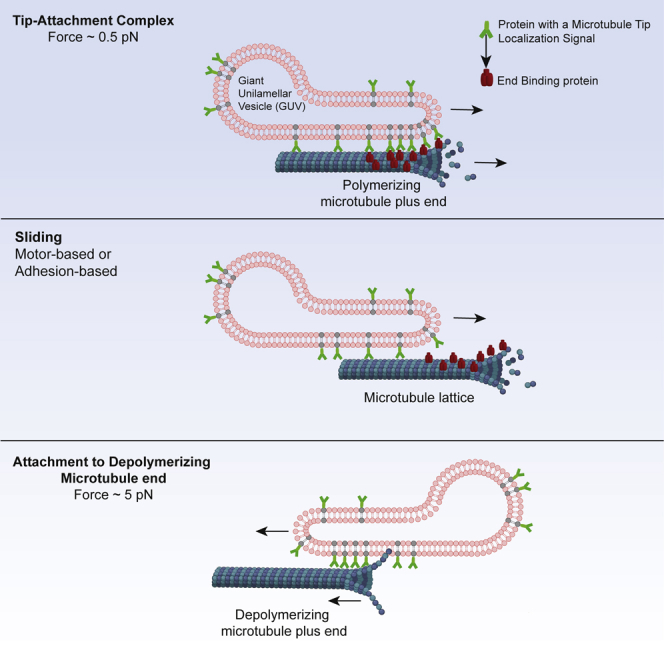

## Teaser

Intracellular organelles can undergo motility by attaching to dynamic microtubule ends, but the underlying biophysical mechanisms were unclear. Rodríguez-García et al. reconstituted membrane remodeling by dynamic microtubules with a minimal set of purified components, measured the associated forces and generated a model describing this process.

## Introduction

**Figure 1:**
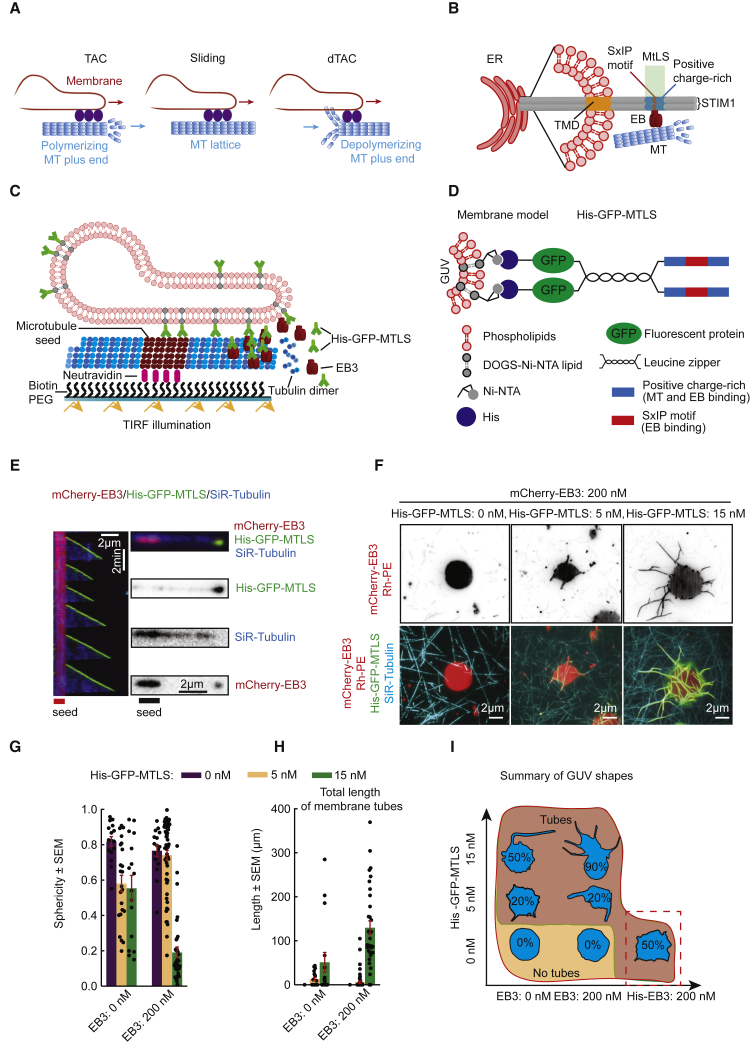
MT-Driven Formation of Membrane Tubes in the Presence of Dynamic MTs, EB3, and His-GFP-MTLS (A) A scheme of the processes implicated in the extension and positioning of ER tubules. (B) A scheme of a TAC formed on the ER membranes by STIM1 and an EB protein. TMD, the transmembrane domain of STIM1. (C and D) Schematic representations of the assay that includes GUVs, GMPCPP-stabilized MT seeds (which contain biotinylated tubulin and are attached to biotin-PEG-coated glass slides through neutravidin), unlabeled tubulin, mCherry-EB3, and His-GFP-MTLS that binds to DOGS-NTA-Ni on the GUV surface (C) and of the His-GFP-MTLS protein (D). (E) Kymographs and snapshots, showing the accumulation of the indicated proteins grown from rhodamine-labeled MT seeds. See also Figures S1A–S1E and Video S1. (F) Representative images of a tubular network observed at different concentrations of His-GFP-MTLS and 200 nM EB3. Each image is the maximum intensity projection of 100 frames. See also Video S2. (G and H) GUV sphericity (G) and the total length of membrane tubes (H), measured at different His-GFP-MTLS and EB3 concentrations. n numbers, from left to right: 16, 23, 16, 16, 57, and 34 GUVs. See also Figure S2. (I) A diagram showing characteristic GUV shapes observed as a function of His-GFP-MTLS and EB3 concentrations. The numbers represent the proportion of tubular GUVs; n numbers from left to right: 0 nM MTLS, 16, 16, and 27, at 5 nM MTLS, 23 and 57, at 15 nM MTLS, 16 and 34.

Microtubules (MTs) are major cytoskeletal filaments, which can generate forces required for many cellular processes. MT-based motors can produce force to position and shape cellular organelles [1]. Furthermore, dynamic MTs generate pushing and pulling forces in a motor-independent fashion [2, 3]. For example, it has been proposed that the attachment of cellular structures to growing MT ends by MT plus-end-tracking proteins (+TIPs) can lead to force generation. The core components of +TIP complexes are End Binding (EB) proteins, which recruit to growing MT ends a large variety of different partners [4]. A force-generating mechanism dependent on EB1 and its tip-tracking partner contributes to chromosome congression during mitosis [5]. Furthermore, the interaction of EB1 and the transmembrane ER protein STIM1 promotes extension of ER tubules [6]. This mechanism of ER remodeling, driven by the membrane-MT tip attachment complex (TAC) (Figure 1A) [6, 7, 8, 9], represents one of the molecular pathways that shape and distribute ER membranes.

The ER morphology ranges from the nuclear envelope to dynamic tubules. Formation of highly curved membranes, such as tubules, is energetically unfavorable and requires applied forces [10, 11, 12]. The generation, stabilization, and distribution of curved membranes in cells depend on the lipid composition of the two membrane leaflets, membrane-deforming proteins, and the cytoskeleton [11, 12, 13]. ER tubulation depends on membrane-shaping proteins such as reticulons [14], whereas ER distribution throughout the cell requires MTs [15]. ER tubules can extend along pre-existing MTs in a process termed sliding (Figure 1A), which is usually driven by motors such as kinesin-1 and is the major form of ER motility in cultured cells [6, 8, 16, 17]. However, in some systems like, for example, *Xenopus* egg extracts, where the activity of kinesin-1 is low [18], TAC-based ER tubule extension is the predominant mechanism of peripheral ER tubule extension [19]. +TIP-dependent ER localization also plays a role in neuronal dendrites [20, 21]. In cultured cells, TAC-driven ER extension represents a relatively small fraction of ER movements [6, 9, 16]; however, inhibition of STIM1-EB1-mediated ER-MT attachments during cell division ensures ER exclusion from the mitotic spindle [22].

Whereas extraction of membrane tubes by motors moving on stabilized MTs has been extensively characterized by *in vitro* reconstitution experiments [23, 24, 25, 26, 27], the mechanisms underlying TAC-mediated membrane remodeling are unclear. Cell-biological experiments demonstrated that ER-resident STIM1 accumulates at growing MT plus ends in EB-dependent manner, because it contains an EB-binding MT Tip Localization Signal (MtLS) (Figure 1B) [28]. However, it is unknown whether EBs and STIM1 are sufficient for membrane tubule extension. It is also unclear whether and how various +TIPs, most of which arrive to growing MT tips by diffusion and rapidly exchange at these locations [29, 30], can mediate force generation. Finally, recent work has shown that ER tubules can be pulled by shrinking MT ends, a mechanism termed dTAC [9] (Figure 1A), but the molecular mechanisms underlying dTAC are unknown.

Here, we reconstituted motor-independent membrane tubulation driven by sliding, TAC, and dTAC mechanisms in an *in vitro* system with purified components, measured the associated forces, and generated a model explaining membrane remodeling by adhesion-driven membrane spreading on MTs. We also examined the interplay between motor-based and TAC-based membrane tubulation and found that the two processes can synergize.

## Results

### A Membrane-Bound MtLS-Containing Protein and EB3 Promote Membrane Tube Extension by Dynamic MTs *In Vitro*

To reconstitute *in vitro* the interaction between membranes and dynamic MTs (Figure 1C), we used giant unilamellar vesicles (GUVs) prepared from POPC (94.95%), DOGS-NTA-Ni (5%), and Rh-PE (0.05%). The GUVs were produced by swelling a dried film of lipids in a 300 mM sucrose solution, and the osmolarity of the solution outside of the GUVs was adjusted to 320 mM, so that the GUVs had low tension, but no tube formation caused by osmotic stress was observed. MTs were grown from GMPCPP-stabilized MT seeds as described previously [29] in the presence of tubulin, mCherry-EB3, and an engineered protein termed His-GFP-MTLS (Figure 1D). His-GFP-MTLS contained the C-terminal 43 residues of human MT-actin cross-linking factor 2 (MACF2) bearing an EB-binding MtLS, which consists of the four-amino-acid-long motif SxIP embedded in an intrinsically disordered, positively charged region [28]. His-GFP-MTLS included a 6-Histidine tag (His) for the interaction with the Ni-NTA moiety on the GUV surface and was dimerized via GCN4 leucine zipper (Figure 1D). As described previously [28], His-GFP-MTLS is representative of other EB-dependent +TIPs, such as STIM1, which is also a dimer with a single SxIP motif embedded in a positively charged sequence exposed in the cytoplasm [31] (Figure 1B).

In the assay with dynamic MTs, His-GFP-MTLS was strongly enriched at growing MT ends in an EB3-dependent manner, and its accumulation at MT tips increased at higher EB3 concentrations (Figures 1E and S1A–S1E; Video S1). Furthermore, His-GFP-MTLS weakly bound along MT shafts, likely because MTs are negatively charged and His-GFP-MTLS is positively charged; this binding was EB3 independent. Whereas in the absence of GUVs the number of His-GFP-MTLS molecules at the MT tip remained constant, it decreased over time when GUVs were included in the assay (Figure S1D). This indicates that His-GFP-MTLS molecules were gradually recruited to the GUV surface and could mediate the interaction between GUVs and MTs.

Video S1. *In Vitro* Plus-End Tracking of His-GFP-MTLS in the Presence of EB3, Related to Figure 1 The sample was prepared with 15 nM His-GFP-MTLS, 50 nM EB3 (left) and 200 nM EB3 (right). Images were acquired sequentially using TIRF microscopy with a 3 s interval. The video is displayed at 15 fps. Scale bar, 5 μm.

When GUVs were combined with MTs, within a few minutes, membrane tubes started to extend along SiR-tubulin-labeled MTs (Figures 1F, S2A, and S2B). To characterize membrane morphology, we measured GUV sphericity and the total length of all tubes in the network [25] (Figures 1G, 1H, and S2B). In the absence of His-GFP-MTLS, GUVs preserved their round shape even at 200 nM EB3 (Figures 1F–1I). In contrast, in the presence of His-GFP-MTLS alone, some tubes were present, and their number increased with the His-GFP-MTLS concentration (Figures 1F, 1H, and 1I). Short tubes could be detected at 200 nM EB3 and 5 nM His-GFP-MTLS; however, only when the concentration of His-GFP-MTLS was increased to 15 nM, extensive tubulation of 90% of the GUVs was observed (Figures 1F–1I).

To test whether MT-membrane interactions can be supported by EB3 alone, we used its 6-Histidine-tagged version (His-EB3), which could directly interact with growing MT tips and DOGS-NTA-Ni. However, even at 200 nM His-EB3, only short tubular structures were present (Figures 1I, S2C, and S2D). Reducing ionic strength of the buffer to promote EB3 interactions with MTs did not change this outcome (Figure S2D). These results indicate that an EB protein directly linked to membranes can induce some membrane tubulation, but the generation of long membrane tubes occurs much more efficiently when an EB protein is combined with a MT-binding, membrane-attached partner.

### MT-Dependent Membrane Tubulation Is Limited by Tension

**Figure 2:**
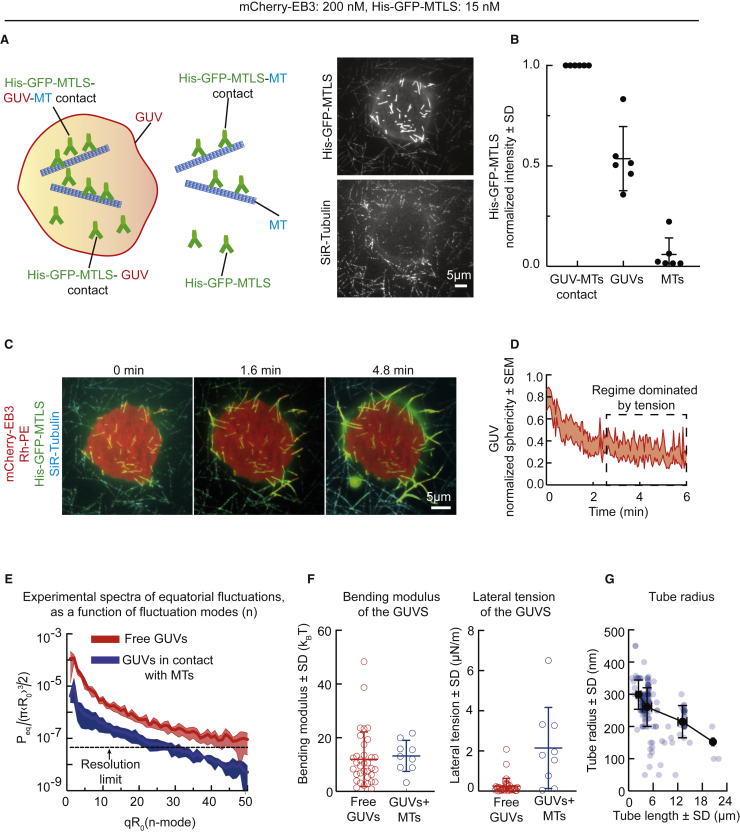
Control of MT-Dependent Formation of Tubular Membrane Networks by Adhesion and Tension (A) Shown on the left is a schematic representation of different binding states of His-GFP-MTLS in the assay. Shown on the right is a snapshot of a time-lapse video showing free and GUV-bound MTs. (B) Averaged MT intensities of the His-GFP-MTLS channel for GUV-attached MTs (n = 6 videos, 62 MTs), the region of the GUV free of MTs (n = 6 videos, 62 profiles), and free MTs (n = 6 videos, 106 MTs). The values were normalized to the mean value of the GUV-attached MTs. (C) Snapshots of a time-lapse video showing the development of a tubular membrane network over time. See also Video S2. (D) Averaged GUV sphericity as a function of time, normalized to the maximum experimental value. The shaded area represents SEM (n = 11). (E) Experimental fluctuation spectrum calculated from the time averages of quadratic fluctuation amplitudes of the equatorial modes, for free vesicles (n = 39) and vesicles in contact with MTs (n = 13), as a function of the fluctuation mode *n*. The dashed line represents the resolution limit, estimated by measuring the fluctuation spectrum of a fixed object in the focal plane. Shaded areas represent SEM. See also Figure S3. (F) Estimated values of the bending modulus and lateral tension for free GUVs (n = 38) and GUVs in contact with MTs (n = 10). (G) Tube radius as a function of tube length. Blue dots, experimental data; black dots, mean values of the radius inside intervals of 4 μm. See also Figure S3D.

Previous studies have shown that ligand-dependent adhesion of GUVs to the surface of a solid substrate triggered membrane tubulation [32, 33] and that mobile ligands on the GUV surface moved to the contact region and increased adhesion strength [34]. In agreement with these findings, His-GFP-MTLS was enriched in the regions where GUVs interacted with MTs (Figures 2A and 2B). His-GFP-MTLS thus converged toward the sites of MT-membrane contacts, suggesting an increase in the number of bonds between the two structures.

Next, we investigated tubulation dynamics by measuring membrane sphericity and found that at the beginning of the assay, the network of membrane tubes rapidly expanded (Figures 2C and 2D). At later time points, sphericity became almost constant as tube extension slowed down (Figures 2C and 2D; Video S2). We hypothesized that this was caused by the increase in lateral tension after the excess of membrane area redistributed into tubes [35, 36]. To test this possibility, we characterized thermal membrane fluctuations by flickering spectroscopy [10, 37] (Figures 2E and S3A–S3C). In the presence of MTs, the amplitude of membrane fluctuations was reduced by an order of magnitude (Figure 2E). The elastic modulus that we measured (Figure 2F) was similar in both conditions and in agreement with previously published values [37]. Importantly, lateral membrane tension was significantly higher in the presence of MTs, leading to the reduction in the fluctuation amplitude (Figures 2E and 2F). We also determined the radius of the tubes by using stimulated emission depletion (STED) microscopy and found that it was in the range of 50–450 nm (Figures 2G and S3D). Longer tubes were thinner (Figure 2G), as can be expected if the tension increases with the tube length [38]. Taken together, these data indicate that GUV extension along MTs follows previously described membrane adhesion dynamics [35, 39], where the initial spreading is counteracted by lateral membrane tension.

Video S2. Membrane Tubes Pulled from a GUV in the Presence of MTs, Related to Figure 1 Images of Rh-PE (red, membrane), GFP (green, His-GFP-MTLS), mCherry-EB3 (red) and SiR-tubulin (cyan, MT) were acquired sequentially using TIRF microscopy with a 3 s interval. The video is displayed at 15 fps. Scale bar, 5 μm.

### Membrane Remodeling Occurs by Three Mechanisms

**Figure 3:**
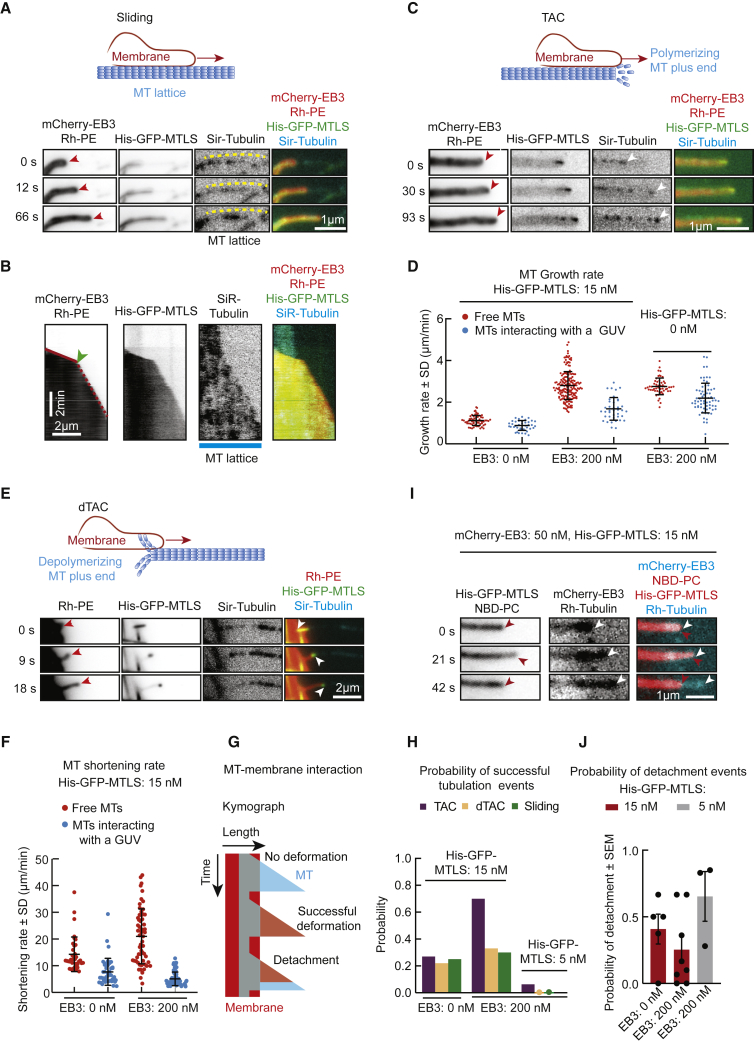
Three Mechanisms of MT-Induced Membrane Tube Formation (A and C) Time-lapse images of a membrane tube (red arrowheads) sliding along a MT (dashed yellow line) (A) or moving together with the plus end of a growing MT (C) (white arrowheads) in the presence of 200 nM mCherry-EB3 (A), 50 nM mCherry-EB3 (C), and 15 nM His-GFP-MTLS. See also Figures S4A, S4C, S4D, S4F, and S4G and Videos S3, S4, and S6. (B) Kymograph of a membrane tube sliding along a MT and catching up with the MT tip (green arrowhead). (D) Growth rate of free MTs and MTs in contact with a membrane; n, from left to right: 63, 41, 179, 40, 41, and 67. (E) Time-lapse images of a membrane tube (red arrowhead) attached to the plus end of a depolymerizing MT (white arrowhead) (dTAC) in the presence of 15 nM His-GFP-MTLS without mCherry-EB3. See also Figure S4E and Video S5. (F) Shortening rate of free MTs and MTs pulling a membrane; n, from left to right: 36, 41, 58, and 73. (G) A scheme of a kymograph showing membrane deformation by a dynamic MT and membrane detachment from the tip. (H) Probability of successful membrane tube generation events occurring through different mechanisms, determined by dividing the number of observed tubes by the total number n of MT-membrane contacts that could potentially support a specific event. Numbers of events and experiments, from left to right, 48 (7), 36 (5), 50 (5), 68 (9), 6 (5), 30 (5), 37 (3), 20 (3), and 41 (3). (I) Snapshots of a video showing a membrane tube (red arrowheads) detaching from a MT tip (white arrowheads). (J) Probability of detachment events from a MT tip. n number of experiments (number of events), from left to right: 7 (50), 9 (68), and 3 (37).

Detailed investigation of membrane dynamics showed that some membrane tubes slid along MT shafts with a rate that was faster than MT elongation rate and slowed down after reaching a growing MT end, beyond which they could not extend (Figures 3A–3D, S4A, and S4B; Video S3). A growing MT end could also initiate tube formation that did not involve prior membrane sliding along a pre-existing MT (Figure S4C; Video S4). As before, in the presence of EB3, His-GFP-MTLS was enriched at MT tips (Figures 3C and S4D; Video S4). Interestingly, the contact of a MT end with a membrane correlated with a decreased MT growth rate (Figure 3D). Given that a similar effect was observed in the absence of His-GFP-MTLS (Figure 3D), this effect was not due to the direct membrane-MT interaction and was possibly caused by the decreased tubulin diffusion to the MT tip.

Video S3. Membrane Tube Sliding along a MT, Related to Figure 3 Images of Rh-PE (red, membrane), GFP (green, His-GFP-MTLS), mCherry-EB3 (red) (left) and SiR-tubulin (MT) (right) were acquired sequentially using TIRF microscopy with a 3 s interval. The video is displayed at 15 fps. Scale bar, 2 μm.

Video S4. Membrane Tube Extending Together with a Growing MT Plus End, Related to Figure 3 Images of Rh-PE (red, membrane), GFP (green, His-GFP-MTLS), mCherry-EB3 (red) (left) and SiR-tubulin (MT) (right) were acquired sequentially using TIRF microscopy with a 3 s interval. The video is displayed at 15 fps. Scale bar, 2 μm.

Recent work demonstrated that in cells, ER tubules can be pulled by depolymerizing MT ends (dTAC) [9]. Also in our experiments, shrinking MTs could extract long membrane tubes from GUVs (Figures 3E and S4E; Video S5). At the tips of such membrane tubes, we observed increased accumulation of His-GFP-MTLS but not of EB3 (Figures 3E and S4E; Video S5). The shortening rate of MTs that were in contact with a membrane was reduced ∼3-fold compared with free MTs (Figure 3F), in agreement with the fact that protein complexes capable of following shortening MT ends can slow down MT depolymerization [40, 41]. Shortening MT ends are characterized by rapid disassembly of tubulin protofilaments, which bend outward, generate a power stroke, and, when connected to cargo by MT-binding proteins, can cause cargo displacement [42]. Our results suggest that membrane-bound His-GFP-MTLS proteins accumulating at the MT-membrane interface create an attachment site that can transmit the force generated by a shortening MT to the membrane.

Video S5. Membrane Tube Pulled by a Depolymerizing MT, Related to Figure 3 Images of Rh-PE (red, membrane) and GFP (green, His-GFP-MTLS) (left) and SiR-tubulin (MT) (right) were acquired sequentially using TIRF microscopy with a 3 s interval. Note the strong accumulation of the His-GFP-MTLS at the tip of the membrane deformation. The video is displayed at 15 fps. Scale bar, 2 μm.

Next, we estimated the probability of successful membrane tubulation events (Figure 3G) occurring through sliding, TAC, and dTAC mechanisms at the membrane contacts with either MT shafts, growing or shrinking MT ends, respectively. In the absence of EB3, the probability was similar for all three mechanisms (Figure 3H), as expected, because all events depended exclusively on the membrane-MT linkages mediated by His-GFP-MTLS and were insensitive to the state of the MT end. However, whereas the probability of successful sliding and dTAC events was not affected by EB3, the probability of successful TAC events was much higher in the presence of EB3 (Figure 3H). This could be explained by the ability of EB3 to concentrate His-GFP-MTLS at growing MT ends and increase the efficiency of a force-generating contact (Figures 1D, 3C, S1A–S1C, and S4D).

The interactions of GUVs with growing MT ends led to short (non-tubular) or long (tubular) membrane deformations (Figures 3C, S4F, and S4G; Videos S4 and S6). These deformations retracted either because a MT depolymerized or because the membrane detached from the growing MT tip, similar to previous observations in cells [6, 7, 8] (Figures 3I, S4F, and S4G; Videos S4 and S6). The fraction of MT-membrane contacts leading to formation of long tubes was higher if both His-GFP-MTLS and EB3 were present and was increased at a higher His-GFP-MTLS concentration (Figure 3H). A higher concentration of His-GFP-MTLS also suppressed membrane tube detachment from MTs (Figure 3J) and thus promoted tube extension. Increased abundance of the complexes formed by EB3 and His-GFP-MTLS at MT tips thus stimulates TAC-mediated membrane tubulation.

Video S6. Extension and Retraction of MT-Dependent Non-tubular GUV Deformations, Related to Figure 3 Images of Rh-PE (red, membrane), mCherry-EB3 (red) and SiR-tubulin (cyan, MT) were acquired sequentially using TIRF microscopy with a 3 s interval. The video is displayed at 15 fps. Scale bar, 3 μm.

### Cooperation between Motor- and TAC-Driven Membrane Tubulation

**Figure 4:**
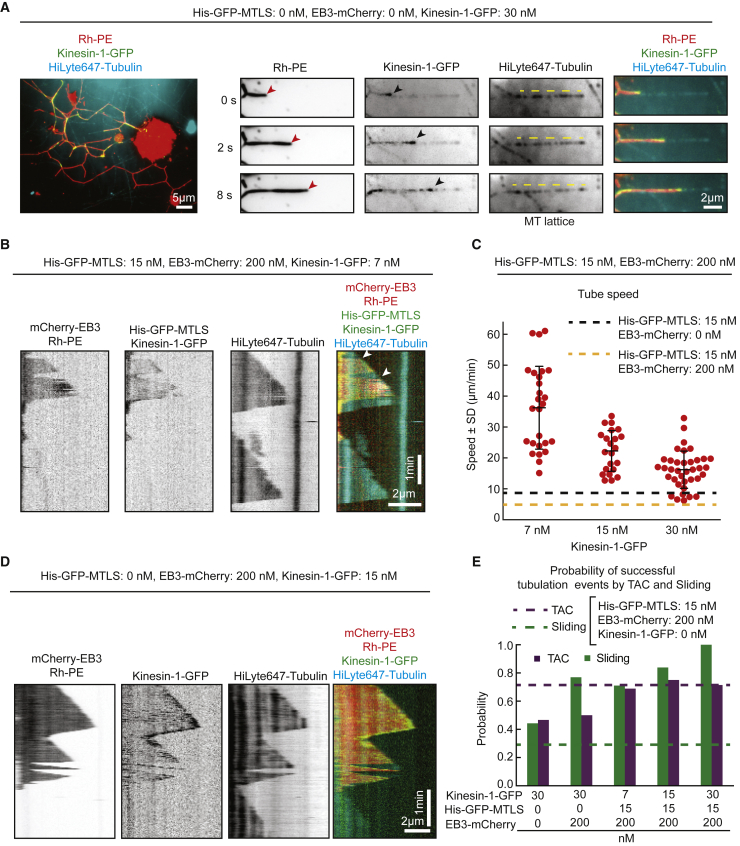
Membrane Tubulation by Membrane-Attached Kinesin-1 (A) Shown on the left is a representative image of a tubular network observed in the presence of 30 nM kinesin-1-GFP. Shown on the right is a time-lapse images of a membrane tube (red arrowheads) pulled by kinesin-1-GFP (black arrowheads) along a MT (dashed yellow line) in the same conditions. (B) Kymograph of a membrane tube sliding along a MT and catching up with the MT tip (white arrowhead) in the presence of 15 nM kinesin-1-GFP, 200 nM EB3, and 15 nM His-GFP-MTLS. (C) Speed of membrane sliding along MTs at 7 nM (n = 35), 15 nM (n = 55), and 30 nM (n = 59) kinesin-1-GFP in the presence of 200 nM EB3 and 15 nM His-GFP-MTLS. The dashed lines represent the mean values of the membrane sliding speed without kinesin (see Figure S4B). (D) Kymograph of a membrane tube moving together with the plus end of a growing MT in the presence of 15 nM kinesin-1-GFP and 200 nM EB3. (E) Probability of the indicated successful membrane tube generation events, determined as in Figure 3H. Numbers of events in 2 experiments, from left to right: 15, 61, 8, 26, 45, 38, 36, 31, 21, and 55.

Given that in cells TAC- and motor-based membrane remodeling typically co-exist, we next tested the effect a membrane-attached kinesin on membrane tubulation in our system. We used a dimeric motile N-terminal fragment of kinesin-1 (residues 1–421 of kinesin heavy chain from *D. melanogaster*), which was fused to GFP and a 6-Histidine tag for attachment to DOGS-NTA-Ni. In agreement with previously published data obtained with stabilized MTs [23, 24, 25, 26, 27], membrane-attached kinesin alone efficiently pulled membrane tubes along MTs and was enriched at the leading tips of extending tubes (Figure 4A). In the presence of His-GFP-MTLS and EB3, kinesin-1-GFP could also efficiently pull membrane tubes, and the speed of their extension was much higher than that observed in the absence of the motor (Figures 4B and 4C). The speed of tube extension was somewhat decreased at higher kinesin-1 concentrations (Figure 4C), possibly because of motor crowding [43]. When the membrane tube reached the growing MT plus end, it could continue extending together with the growing MT tip, while occasionally retracting and extending again (Figure 4B). This membrane behavior did not depend on the presence of His-GFP-MTLS (Figure 4D), indicating that kinesin-1-GFP alone can maintain the connection between the tip of a membrane tube and a dynamic MT end. In the presence of kinesin, the probability of sliding increased, but it could be further increased when EB3 was present (Figure 4E). The simultaneous presence of kinesin, EB3, and His-GFP-MTLS also increased the probability of TAC events (Figure 4E). We conclude that although a kinesin and a +TIP complex can independently induce membrane tubulation, the combined presence of kinesin and +TIP complexes promotes both sliding and TAC-dependent events.

### Modeling of Membrane Spreading Driven by TAC Formation

**Figure 5:**
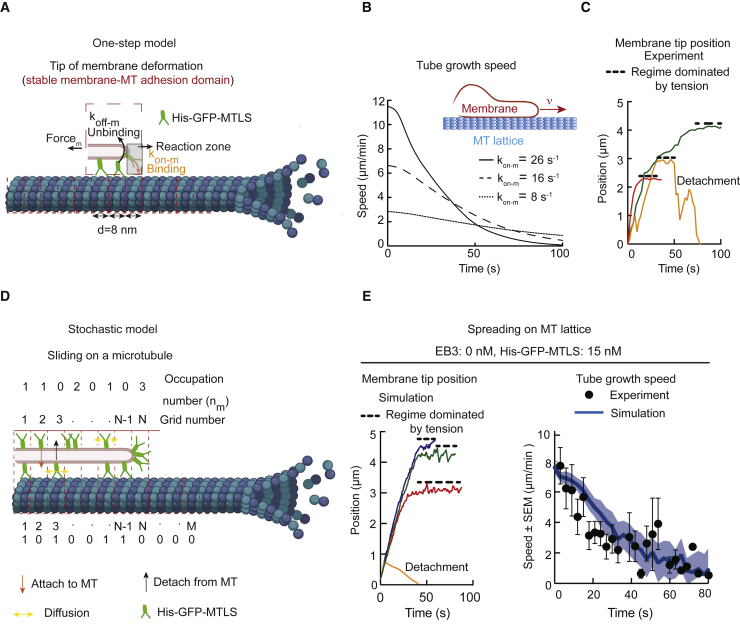
Modeling and Simulations of Membrane Spreading along MT Shafts and Comparison of *In Silico* and Experimental Data (A) Kinetic scheme for the one-step model. (B) Predicted speed of membrane tip spreading along MT shafts as a function of time for three different values of the association rate using Equation 12 (STAR Methods). Curves are averages of 100 individual traces with randomly sampled membrane parameters. (C) Experimental traces of three membrane tube tips sliding along MTs. (D) Kinetic schemes for the simulations of a membrane sliding along a MT shafts. (E) Shown on the left are traces produced by independent simulations of tip positions of membrane tubes sliding along MTs. Shown on the right is the average tube velocity as a function of time (blue), estimated from 60 simulations of membrane spreading on MTs. The shaded region represents SEM. Black dots, experimental values of the average tube velocity (n = 12). See also Figures S5A and S5B and Table S1.

We next set out to obtain a quantitative description of membrane spreading process by combining theory with experimental measurements of different parameters of membrane-MT interactions (Table S1). We first described the membrane sliding mechanism by using a simple analytical model [44] (see STAR Methods). We assumed that the interaction between GUVs and MTs is dominated by the binding of the His-GFP-MTLS to MTs. We modeled the formation of a fixed-size membrane-MT adhesion domain (Figure 5A). The interactions between His-GFP-MTLS and MTs were described by a binding rate *k*_*on-m*_ (Table S1, parameter 4) and a detachment rate *k*_*off-m*_ (Table S1, parameter 3), which were calculated from the experimentally measured values (Table S1; Figures 5A and S5A–S5F).

In this model, the highest initial speed V of membrane spreading depends on the association rate *k*_*on-m*_ and force-dependent detachment rate *k*_*off-m*_*(F*_*m*_*)* (Figure 5A; see Equation 12 in STAR Methods). We assumed that, as the membrane tube increases in length, the force needed to pull a tube also increases, and therefore the tube elongation rate slows down and finally drops to a value close to zero (Figure 5B). This final state matches well the saturation regime observed experimentally (Figure 5C).

Next, we extended our simple model by taking into consideration the formation of bonds not only at the membrane tube tip but also all along the contact interface between the membrane deformation and a MT (Figures 5D and S5G–S5I) [23]. Due to the high number of interactions to be considered, we performed *in silico* experiments using a stochastic approach [45, 46] (see STAR Methods). When the EB3 concentration is zero (Figures 5D and 5E), the estimated on-rate of His-GFP-MTLS is *k*_*on-m*_ = 16 s^–1^ (Table S1, parameter 4). In these conditions, simulations resembled the experimental observations quite well (Figures 5C and 5E). Furthermore, the saturation regime observed in experiments and simulations (Figures 5C and 5E, left panel) indicates that the tension increases with the tube length. Such increase depends on the initial tension and the size of the membrane. Both values define a threshold length that separates a negligible tension regime from the non-negligible one. We estimated that for a GUV in the entropic regime (tension = 10^−7^ N/m), with a radius of 10 μm and bending modulus 5 × 10^−20^J, the threshold tube length is approximately 1–2 μm. This estimation matches well with the saturation regime observed in our experiments (Figures 5C and 5E, left). The predicted sliding speed overlapped quite well with the experimental data (Figures 5E, right). In addition, the estimated membrane spreading speeds from simulations and experiments agreed with the predictions of the analytical model, showing a decay that was dominated by tension (Figures 5B and 5E, right).

**Figure 6:**
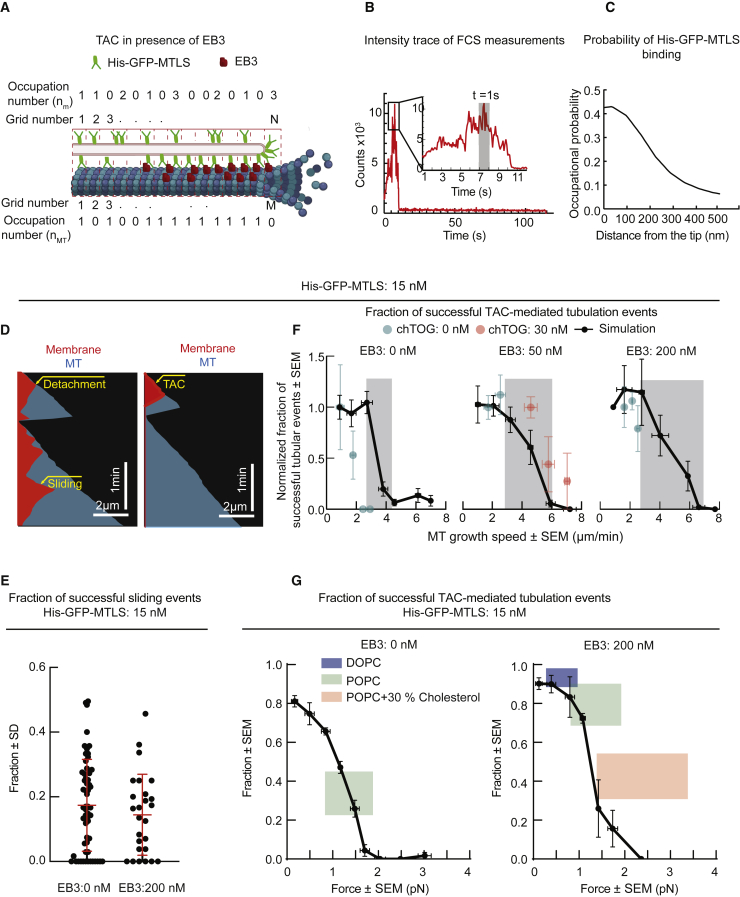
Simulations of TAC-Based Membrane Tubulation and Comparison of *In Silico* and Experimental Data (A) Kinetic scheme for the simulations of a membrane sliding at the MT tip. (B) Representative intensity time trace of a MT tip labeled with EB3-GFP recorded by FCS. The inset shows maximum intensity during a time window of 12 s. The gray rectangle marks the intensity in 1 s. (C) Estimated occupational probability of His-GFP-MTLS at the MT tip in the presence of 200 nM EB3. (D) Kymographs obtained from the stochastic simulations of membrane spreading in the presence of dynamic MTs. (E) Fraction of successful sliding-driven tubulation events, calculated from simulations in the same way as in Figure 3H. n, number of independent simulations (black dots) EB3 0 nM, 58 (1,007 traces); EB3 200 nM, 28 (348) traces. (F) Fraction of membrane deformations becoming tubular as a function of MT growth rate in simulations and experiments. Data were normalized to the average fraction obtained at the lowest MT growth rate. Gray rectangles mark the areas where the probability decays with the speed. n, number of events experiments: 0 nM EB3, 50 (7); 200 nM EB3, 68 (9); 50 nM EB3, 28 (7); 30 nM chTOG; and 50 nM EB3, 22 (4). Simulations: EB3 0 nM, 17 (87 traces); EB3 50 nM, 57 (1510 traces); EB3 200 nM, 28 (348 traces). Every experimental point represents the mean speed, calculated from several individual experiments inside intervals of 0.5 μm/s. (G) Fraction of membrane deformations becoming tubular as a function of the force needed to pull a tube. n, number of experiments: POPC, EB3 0 nM and His-GFP-MTLS 15 nM, 48 (7); POPC, EB3 200 nM and His-GFP-MTLS 15 nM, 68 (9); POPC+30% cholesterol, EB3 200 nM and His-GFP-MTLS 15 nM, 61 (3); DOPC, EB3 200 nM and His-GFP-MTLS 15 nM, 22 (2). Simulations: EB3 0 nM, 59 (824 traces); EB3 200 nM, 31 (424 traces). The experimentally measured values are represented by rectangles due to the experimental uncertainty. See also Table S1.

Finally, we incorporated into the model the EB3-induced enrichment of His-GFP-MTLS on MT tips compared with MT shafts (Figure 6A). TAC formation can be regarded as the assembly of an EB3-dependent adhesion domain at MT tips, with the *k*_*on-m-tip*_ of His-GFP-MTLS at the MT tip being 2.5 higher than *k*_*on-m*_ of His-GFP-MTLS at MT lattice (Table S1, parameter 5) (Figures 6A–6C). We used fluorescence correlation spectroscopy (FCS) to estimate the maximum number of His-GFP-MTLS molecules at MT tips (Figure 6B). We then estimated the probability of His-GFP-MTLS binding along MT shafts (Figure 6C), using experimentally measured His-GFP-MTLS intensity profiles (Figure S1B). In *in silico* experiments, simultaneous movements of the membrane and MT tips were classified as TAC events, whereas other membrane spreading events were classified as sliding (Figure 6D). Similar to the experimental data (Figure 3H), the probability of successful sliding events observed in the simulations did not depend on the presence of EB3 (Figure 6E).

The analytical model predicted that there was a certain maximum speed at which a membrane could slide along a MT (Figure 5B and Equation 12 in STAR Methods). Therefore, the TAC-driven membrane tubulation could be impeded at high MT growth rates. In agreement with this idea, the simulations predicted that the fraction of successful tubular deformations was approximately constant when MTs grew with a speed of 1–3 μm/min, but, when MT growth rates were increased, the system approached a transition regime where the probability of tubular deformations rapidly decayed, indicating that the formation of a TAC complex was limited by the maximum sliding speed of the membrane (Figure 6F). The inclusion of EB3 in the simulation increased the fraction of TAC-mediated membrane tubulation at higher MT growth rates, because of a higher *k*_*on*_ and thus higher maximum spreading speed (Figure 6F). Also in our experimental system, in the absence of EB3, the transition to low tubulation probabilities was found at lower MT growth speeds than in the presence of EB3, indicating that EB3 enhanced the capacity of the membrane to follow the MT tip (Figure 6F). We note that the results of the simulations matched the experimental data in the presence of EB3 better than in the absence of EB3 (Figure 6F).

Given that MT growth speeds in our system were below the range where, according to the simulations, the transition to low tubulation probability occurred, we increased MT growth speed by adding the human MT polymerase chTOG [47]. In the presence of chTOG, MT polymerization rate was indeed increased, whereas membrane tubulation probability was reduced, just as our simulations predicted (Figure 6F). Taken together, these results support the idea that TAC formation is limited by the MT growth rate.

### Estimation of Forces Sustained by TACs during Membrane Spreading

To estimate the forces that TACs could sustain, we performed stochastic simulations at different values of the force needed to pull a membrane tube [38] and estimated the probability of TAC formation in GUVs at different forces (Figure 6G). To compare our simulations with experiments, we varied the GUV rigidity by either using DOPC instead of POPC to reduce membrane stiffness or by adding cholesterol, to make membranes stiffer [37, 48]. As expected [23, 25, 38], the probability of tubulation decreased with an increase in the force required to make a tube. We estimate the maximal force above which no tubes could be pulled by spreading as ∼1.5–2 pN, depending on the EB3 concentration, with the fraction of successful tubulation events at these forces being ∼5%. At the same lipid composition and thus the same membrane rigidity, the presence of EB3, which promotes TAC assembly, increased the chances of pulling a tube both in the simulations and in the experiments (Figure 6G). Altogether, our simulations, which are based on experimentally measured parameters and incorporate known physical effects related to membrane mechanics, provide a good quantitative explanation of the experimentally observed features of our reconstitution system.

### Measurement of TAC-Mediated Force Production

**Figure 7:**
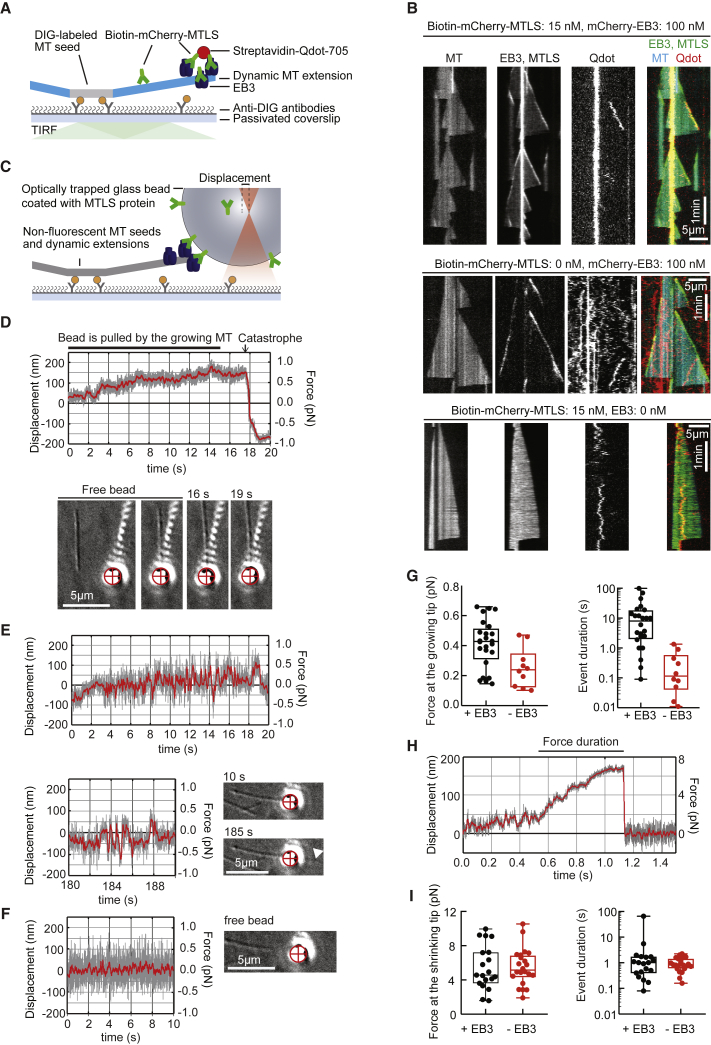
Cargo Transport and Force Generation by Growing and Shrinking MT Tips (A) Schematics of the experimental setup. (B) Kymographs showing MTs polymerized from HiLyte-488 tubulin, bio-mCherry-MTLS, and mCherry-EB3 and Qdot705-streptavidin. Notation above the kymographs describes the experimental conditions. See also Video S7. (C) Setup of the optical trap experiments. (D) An example of a bead trace in the presence of EB3 showing the raw signal at 1 kHz (gray) and the signal after smoothing with a running average of 100 points (red). Images at the bottom show corresponding frames from the DIC video (Video S8). The red cross marks the center of the optical trap. (E) Analogous experiment in the absence of EB3. Bottom trace: the fast back and forth movements continued after the MT tip grew past the position of the trapped bead (white arrowhead). (F) Example trace and image of a free, unattached bead in a trap. (G and I) Force amplitude and force duration measured at the growing (G) or shrinking (I) MT tip, in the presence (black, n = 24 [G] or 19 [I]) or absence (red, n = 10 [G] and 22 [I]) of 100 nM EB3. Horizontal line: median; box: 25%–75%, whiskers: minimal and maximal values. (H) Example trace of a Bio-mCherry-MTLS-coated bead in the absence of EB3 pulled by a shortening MT tip depicted as in (D).

To get further insight into force generation in our system, we first asked whether a quantum dot (Qdot) could be transported by adhesive interactions with growing MT tips without an external load. For this assay, we used streptavidin-coated Qdots and a biotinylated version of the dimeric His-GFP-MTLS (Bio-mCherry-MTLS) (Figure 7A). When both EB3 and Bio-mCherry-MTLS were present, we observed Qdots traveling steadily with the growing MT tips (Figure 7B; Video S7, left). EB3 alone did not promote Qdot binding to MTs, whereas Bio-mCherry-MTLS alone promoted Qdot binding only to MT shafts, but not to growing tips (Figure 7B and Video S7, right). Diffusion and rapid exchange of EB proteins at MT ends [29, 30] can thus promote processive EB-dependent MT-growth-driven motility of a cluster of EB partners.

Video S7. Qdot Transport with the Tip of the Growing MT, Related to Figure 7 Left panel: Three channels were imaged by TIRF microscopy sequentially with a 1.3 s interval: Qdot705-streptavidin (red), HiLyte488-tubulin (cyan), Bio-mCherry-MTLS (15 nM, green) and mCherry-EB3 (100 nM, green). The corresponding kymograph is shown in Figure 7B. The video is displayed at 15 fps. On the right, Qdots fail to bind and track dynamic MTs in the absence of Bio-mCherry-MTLS. Three channels were imaged by TIRF microscopy sequentially at 1.3 s interval: Qdot705-streptavidin (red), HiLyte488-tubulin (cyan) and mCherry-EB3 (100 nM, green). The video is displayed at 15 fps. Scale bars, 5 μm.

Next, we adapted the assay for direct measurement of the force sustained by TACs at growing MT ends. Neutravidin-coated glass beads were incubated with Bio-mCherry-MTLS and added to a chamber containing dynamic MTs, EB3, and Bio-mCherry-MTLS. A bead was optically trapped by using low-power laser tweezers (stiffness ∼0.005 pN/nm) and placed in front of a growing MT tip (Figure 7C). Upon binding to a MT, the bead was initially pulled away from the trap center and then gradually returned to zero position under an assisting load applied by the optical trap (Video S8). When both EB3 and Bio-mCherry-MTLS were present, the beads were displaced by growing MT tips past the zero-force position against the opposing load applied by the optical trap for seconds (Figure 7D). This displacement lasted until a MT catastrophe when the beads that presumably retained their association with the MT tip were pulled out of the trap by the force generated by MT shortening (Figure 7D).

Video S8. A Growing MT End Pushes a Trapped Glass Bead, Related to Figure 7 A 1 μm glass bead coated with bio-mCherry-MTLS trapped in a soft trap was imaged using DIC microscopy at 15 fps and every 10 frames were averaged. The video is displayed at 15 fps. The quadrant photo detector trace corresponding to this experiment is shown in Figure 7E.

In the absence of EB3, the beads returned to zero-force position and did not continue to move with the growing MT tip but underwent frequent and brief bead motions back and forth along the MT (Figure 7E). These motions were clearly different from normal fluctuations of a trapped bead that was free from any interactions with MTs or glass (Figure 7F). We measured the duration and the amplitude of the first positive bead displacement after the initial return to the zero position. Only forces above 0.1 pN (the resolution of our instrument) were quantified. We found that most events in the absence of EB3 were shorter than 1 s, whereas in the presence of EB3 they lasted for 15 ± 5 s (mean ± SEM, n = 24). The forces generated when both EB3 and Bio-mCherry-MTLS were present were in the range of 0.1–0.7 pN, whereas the forces observed without EB3 were significantly lower (Figure 7G). Our data indicate that TAC formation allows growing MTs to exert adhesion-based forces in the range of 0.5 pN.

To measure the forces that are exerted by dTACs during MT shortening, we performed the same experiment using a much stiffer trap (0.03–0.05 pN/nm). In these conditions, the forces in the direction of MT growth were barely distinguishable against the bead’s thermal fluctuations, whereas the forces generated by shortening MT ends pulled the beads from the center of the trap, followed by detachment (Figure 7H). We observed forces in the range of 2–10 pN that typically lasted for 0.2–2 s, independent of the presence of EB3 (Figure 7I). We have thus reconstituted EB3-dependent force coupling of TACs and EB-independent force coupling of dTACs.

## Discussion

In this study, we reconstituted MT-based membrane tubulation by using a minimal system sufficient to drive formation of membrane tubes by three mechanisms: sliding, TAC, and dTAC, all of which were previously described in cells [6, 7, 8, 9]. Previously, the sliding mechanism was attributed to motor proteins [6, 8, 16], and we confirmed that a membrane-attached motor can efficiently pull membrane tubes *in vitro* [23, 24, 25, 26, 27]. However, our data show that membranes can also spread on MT shafts independently of molecular motors because of the adhesion mediated by a protein that can connect a lipid bilayer to MTs. Thus, motor activity is not strictly required for sliding of membrane tubes along pre-existing MTs. Furthermore, both TAC and dTAC events can be driven by a single protein that can connect membranes to MTs, raising the question about the contribution of such biochemically simple mechanisms to shaping the ER and other membrane organelles in cells.

Although membrane tubes could form through the TAC mechanism in the presence of a MT- and membrane-binding protein alone, the efficiency of membrane tubulation was strongly increased in the presence of EB3, to which this protein can bind. Previous work in cells showed that the formation of the TAC complex requires the interaction between the MtLS-containing transmembrane ER protein STIM1 and EBs [6], but it remained unclear whether additional molecules are needed. Here, we showed that a combination of an EB protein and its membrane-associated partner is sufficient to promote efficient membrane tube extension by growing MT ends. This mechanism relies on molecules that, unlike molecular motors, do not undergo processive motion but rather concentrate at growing MT ends by a diffusion-based mechanism because of their affinity for the MT tip structure [29, 30]. Despite their fast turnover on the MT ends, clustering of MT tip-tracking proteins on a bead or a membrane tip is sufficient to bias the displacement of these structures together with the EB comet in the direction of MT growth. EB3 localizes to the growing MT tip in a nucleotide-dependent manner [49], and, although GTP hydrolysis is not required for MT growth, GTP hydrolysis behind the growing MT end is needed for localized high-density binding of EB3 in a form of a comet, which provides directionality to cargo motility in our assays. TAC-dependent processive motility thus exploits the structural asymmetry at the MT plus end induced by GTP hydrolysis. Importantly, the EB3-dependent TAC mechanism was particularly helpful when MTs grew rapidly, and this likely explains its relevance in cells.

Our measurements indicated that in the conditions used, TACs can generate forces in the range of ∼0.5 pN. This value is in line with a recent study that examined the interaction of a MT tip-bound EB with kinesin-14 [50]. Based on the minimal value of tension and the membrane bending modulus, a force in the range of ∼0.5 pN is sufficient to cause tubulation of membranes with very low tension (∼10^−7^ N/m). However, based both on our simulations and experiments, the forces generated during TAC events when membranes were under some tension were in the range of 1–2 pN. The additional force is likely sustained by membrane adhesion along the MT lattice, whereas this contribution is absent in the bead-based assay. Furthermore, the geometry of the membrane-MT interface, with the membrane partly wrapping around the MT cylinder, might lead to a higher number of contacts that can sustain higher forces compared with a MT interface with a spherical bead.

We observed that the presence of a protein with dual affinity to membranes and MT shafts was sufficient to pull membrane tubes by shrinking MTs (dTAC mechanism). Contrary to MT growth, MT shortening depends on GTP hydrolysis and can thus be considered an active process, which can produce forces that were estimated to be in the range of tens of pN [51]. The bead-bound MTLS protein in our system was able to capture a good fraction of this force. Recent imaging work revealed dTAC-based ER remodeling in cells, and it was proposed that this mechanism employed factors different from those specific for TAC generation [9]. However, our data show that the same molecules can in principle support both TAC and dTAC mechanisms. Interestingly, the forces generated when the same MT-membrane connecting molecules are attached to shrinking MTs are an order of magnitude higher than those mediated by TACs, likely reflecting the difference in the amount of force produced by these two mechanisms. We hypothesize that any diffusible ER-resident protein with a strong affinity for MT lattices might be able to induce dTAC formation, if it can accumulate at the interface between the membrane and a depolymerizing MT end. Even a completely artificial peptide can produce coupling to shortening MTs [52], providing validation for this hypothesis.

To conclude, our data show that the three different mechanisms of MT-driven membrane tube extension observed for the ER in cells can be supported by the formation of transient bonds between MTs and membranes and that a biased self-spreading of the lipid bilayer on MTs can account both for the sliding and TAC mechanisms. Whereas previous work has provided the proof of principle for membrane spreading on fibers [33, 53], these studies did not aim to reconstitute molecular mechanisms that are operational in the cell. In contrast, the system used here recapitulated the dynamics and affinities found in naturally occurring MT-binding proteins and dynamic MTs. It is therefore of direct relevance to the MT-membrane contacts found in cells and might help to understand the contribution of MT tip attachment to the morphogenesis of other organelles such as, for example, mitochondria [54] and recycling endosomes [55]. Given that numerous non-motor MT-membrane linking proteins are known, they are likely to contribute to intracellular organelle organization through adhesion-based mechanisms described in our study.

## STAR★Methods

### Key Resources Table

**Table:** 

REAGENT or RESOURCE	SOURCE	IDENTIFIER
**Antibodies**		

Anti-digoxigenin (sheep)	Roche	11333089001

**Bacterial and Virus Strains**		

E.coli BL21 (DE3)	Agilent	200131

**Chemicals, Peptides, and Recombinant Proteins**		

cOmplete™, EDTA-free Protease Inhibitor Cocktail	Roche	Cat#4693116001
cOmplete His-Tag Purification Resin	Roche	Cat#5893682001
Affinity column: 5mL HiTrap IMAC HP	GE Healthcare	Cat#17092005
Size exclusion chromatography: HiLoad 16/600	GE Healthcare	Cat#28989335
StrepTactin Sepharose High Performance	GE Healthcare	Cat#28-9355-99
Tubulin Porcine	Cytoskeleton	Cat#T240-C
Tubulin Porcine TRITC	Cytoskeleton	Cat#TL590M
Tubulin Porcine HiLyte 488™	Cytoskeleton	Cat#TL488M
Tubulin Porcine HiLyte 647™	Cytoskeleton	Cat#TL670M
Tubulin Porcine	Cytoskeleton	Cat#T333P
GMPCPP	Jena Biosciences	Cat#NU-405L
GTP	Sigma	Cat#G8877
Glucose oxidase	Sigma	Cat#G7141
Catalase	Sigma	Cat#C9322
d-Desthiobiotin	Sigma	Cat#D1411
DTT	Sigma	Cat#R0861
k-casein	Sigma	Cat#C0406
Neutravidin	Invitrogen	Cat#A-2666
His-mCherry-EB3 full length	[56]	N/A
mCherry-EB3 full length	This study	N/A
Kinesin-1-GFP	[57]	N/A
Human ch-TOG	This study	N/A
His-GFP-MTLS	This study	N/A
Biotin-mCherry-MTLS	This study	N/A
1-palmitoyl-2-oleoyl-glycero-3-phosphocoline (POPC)	Avanti Polar Lipids	CAT#850457
1,2-dioleoyl-sn-glycero-3-[(N(5-amino-1-carboxypentyl)iminodiacetic acid)succinyl](nickel salt) (DOGS-Ni-NTA)	Avanti Polar Lipids	CAT#790404
1,2-dioleoyl-sn-glycero-3-phosphoethanolamine-N-(lissamine rhodamine B sulfonyl) (ammonium salt) (Rh-PE)	Avanti Polar Lipids	CAT#810150
1,2-dioleolyl-sn-glycero-3-phosphocoline (DOPC)	Avanti Polar Lipids	CAT#850375
1-oleoyl-2-{6-[(7-nitro-2-1,3-benzoxadiazol-4-yl)amino]hexanoyl}-sn-glycero-3-phosphocoline (NBD-PC)	Avanti Polar Lipids	CAT#810132
Cholesterol	Sigma Aldrich	CAT#8667
Poly-L-lysine (20 kDa) grafted with polyethyleneglycole (2 kDa), unlabelled or biotinylated	SuSoS AG	PLL(20)-g[3.5]- PEG(2)
Repel-silane ES	Sigma Aldrich	GE17-1332-01
Qdot 705 Streptavidin Conjugate	Thermo Fisher	Q10161MP
Carboxyl silica beads, 1 μm	Bangs Laboratories	SC04000

**Experimental Models: Cell Lines**		

Human: HEK293T	ATCC	CRL-11268

**Oligonucleotides**		

Oligonucleotides used to clone 6His-3C-mCherryEB3	This study	N/A
Forward primer:		
CTCTTTCAGGGACCCATGGCTAGGCTACCGGTC		
Oligonucleotides used to clone 6His-3C-mCherryEB3	This study	N/A
Reverse primer		
TTGCTAAGTGAGCTCTGTCAATTATCAGTACTCGTC CTGGTCTTCTTGTTGATGC		
Oligonucleotides used to clone Bio-mCherry-MTLS	This study	N/A
Forward primers:		
Bio-mCherry		
GCAAATGGGTCGCGGATCCGAATTCATGGCTTC CGGCCT		
GAAC		
MTLS		
CTCGGCATGGACGAGCTGTACAAGCGTATGAAA CAGCTG		
GAAGACAAAG		
Oligonucleotides used to clone Bio-mCherry-MTLS	This study	N/A
Reverse primers:		
Bio-mCherry		
CTTTGTCTTCCAGCTGTTTCATACGCTTGTACAG CTCGTCC		
ATGCC		
MTLS		
CGAGTGCGGCCGCAAGCTTGTCGACTCATCTC TTTGAGG		
ACTTGTCCAATTTG		

**Software and Algorithms**		

Fiji	NIH	https://imagej.net/Fiji
Metamorph	Molecular Devices	https://moleculardevices.com/products/cellular-imaging-systems/acquisition-and-analysis-software/metamorph-microscopy
MATLAB	Mathworks	https://mathworks.com/products/matlab
Python	Python	https://www.python.org/
GraphPad Prism	GraphPad Software	https://www.graphpad.com/scientific-software/prism/
Huygens Professional software	Scientific Volume Imaging (SVI)	https://svi.nl/Huygens-Professional
KymoResliceWide plugin	Eugene Katrukha	https://github.com/ekatrukha/KymoResliceWide
Simulation Figures 5 and 6	This study	https://github.com/RuddiRodriguez/Spreading;https://github.com/RuddiRodriguez/Spreading_membrane-MT_reaction_inside_EB
Flickering spectroscopy	This study	https://github.com/RuddiRodriguez/programnnn
SymPhoTime	PicoQuant	https://www.picoquant.com/products/category/software/symphotime-64-fluorescence-lifetime-imaging-and-correlation-software

**Other**		

(ITO)-coated glass slides	Sigma Aldrich	CAT#636916
Non-adhesive silicone spacer	Sigma Aldrich	CAT#GBL664304

### Lead Contact and Materials Availability

Further information and requests for resources and reagents should be directed to and will be fulfilled by the Lead Contact, Anna Akhmanova (a.akhmanova@uu.nl). All unique/stable reagents generated in this study are available from the Lead Contact without restriction.

### Experimental Model and Subject Details

#### Bacterial strains

*E.coli* expression strain BL21(DE3) was used for recombinant expression of mCherry-EB3 and Bio-mCherry-MTLS, which were used for the *in vitro* reconstitution assays. The cells were cultured in Luria-Bertani (LB) medium.

#### Human cell lines

Human chTOG construct were overexpressed in HEK293T cells for purification. HEK293T cells were cultured in DMEM/F10 (1:1 ratio, Lonza, Basel, Switzerland) supplemented with 10% fetal calf serum, transfected using polyethylenimine (PEI, Polysciences) and harvested 2 days after transfection. The cell lines used here were not found in the database of commonly misidentified cell lines maintained by ICLAC and NCBI BioSample, were not authenticated and were negative to mycoplasma contamination.

### Method Details

#### Preparation of vesicles

1-palmitoyl-2-oleoyl-glycero-3-phosphocoline (POPC), 1,2-dioleoyl-sn-glycero-3-[(N(5-amino-1-carboxypentyl)iminodiacetic acid)succinyl](nickel salt) (DOGS-Ni-NTA), 1,2-dioleoyl-sn-glycero-3-phosphoethanolamine-N-(lissamine rhodamine B sulfonyl) (ammonium salt) (Rh-PE), 1,2-dioleolyl-sn-glycero-3-phosphocoline (DOPC) and 1-oleoyl-2-{6-[(7-nitro-2-1,3-benzoxadiazol-4-yl)amino]hexanoyl}-sn-glycero-3-phosphocoline (NBD-PC) were purchased from Avanti Polar Lipids. Cholesterol was purchased from Sigma Aldrich. The lipid mixtures were composed of 94.95% POPC, 5% DOGS-Ni-NTA, 0.05% (Rh-PE/NBD-PC) or 94.95% DOPC, 5% DOGS-Ni-NTA, 0.05%Rh-PE. For experiments with cholesterol, the lipid mixture was 64.95% POPC, 5% DOGS-Ni-NTA, 0.05% Rh-PE and 30% cholesterol (expressed as molar proportions). GUVs were prepared by electroformation [58] on two conductor indium tin oxide (ITO)-coated glass slides (Sigma Aldrich). Briefly, ten microliters of a lipid suspension (0.1 mg/mL) in chloroform were spread over the conductor surface. A chamber was made with non-adhesive silicone spacer (0.8 mm depth, Sigma Aldrich); after solvent evaporation the films were hydrated with sucrose solution (300 mM), and the electrodes were connected to an AC power supply (1V, 10 Hz) for 3.5 h and (1.5V, 5 Hz) for 30 min to ensure good detachment of the GUVs from the ITO glass slides. The GUVs were produced in a 300 mM sucrose solution, and for the subsequent assays, the osmolarity of the solution outside of the GUVs was adjusted to 320 mM. In such conditions the GUVs were deflated and the tension was reduced, but since the difference in osmolarity inside and outside of the GUVs did not exceed 10%, there was no membrane tube formation caused by osmotic stress.

#### Protein purification

His-mCherry-EB3 was purified from *E. coli* as described previously [56]. mCherry-EB3 was cloned into a pET-based bacterial expression vector with a N-terminal 6xHis tag followed by a 3C cleavage site using a restriction free positive selection method [59]. Protein production was performed in the *E. coli* expression strain BL21(DE3) in LB broth media by inducing with 0.5 mM IPTG at an OD_600_ of 0.4 to 0.6 over-night at 20°C. Cells were harvested by centrifugation at 4°C, 3,500 x g for 15 min and lysed by sonication in a buffer containing 50 mM HEPES, pH 8.0, supplemented with 500 mM NaCl, 10 mM Imidazole, 10% Glycerol, 2 mM β-mercaptoethanol and protease inhibitors (Roche). The crude extracts were cleared by centrifugation at 20,000 x g for 20 min and the supernatants were filtered through a 0.4 μm filter before purification. Protein purification was performed by immobilized metal-affinity chromatography (IMAC) on HisTrap HP Ni^2+^ Sepharose columns (GE Healthcare) at 4°C according to the manufacturer’s instructions. The 6xHis tag was cleaved overnight using the 3C protease during dialysis against lysis buffer (without protease inhibitors). The cleaved sample was reapplied onto the IMAC column to separate the cleaved product from its tag and potentially uncleaved protein. Processed protein samples were concentrated and further purified on a HiLoad Superdex 200 16/60 size exclusion chromatography column (GE Healthcare) equilibrated in 20 mM Tris HCl, pH 7.5, supplemented with 150 mM NaCl and 2 mM DTT. Protein fractions were analyzed by Coomassie stained SDS-PAGE. Fractions containing mCherry-EB3 were pooled and concentrated by ultrafiltration. Protein concentration was estimated by UV at 280 nm and the pure mCherry-EB3 was aliquoted, flash frozen in liquid nitrogen and stored at −80°C.

His-GFP-MTLS corresponds to the previously described construct that contains EGFP, the two-stranded leucine zipper (LZ) coiled-coil domain of GCN4, and the C-terminal 43 residues of human MACF2 (MACF43) [28]. It was cloned between the *Nde*I and BamHI sites of the pET28a bacterial expression vector (Novagen, Merck Millipore, Billerica, MA, USA) and expressed and purified as described previously [60]. In brief, expression in *E. coli* strain BL21(DE3) was performed in LB medium. Cells were grown at 37°C till an OD600 between 0.6 and 0.8 was reached. After induction with 1 mM IPTG the cells were incubated for 1.5 h at 37°C. The protein was purified at 4°C by immobilized affinity chromatography using Ni-Sepharose columns (Invitrogen) followed by size exclusion chromatography (Superdex 75; GE Healthcare) in 20 mM Tris-HCl, pH 7.5, supplemented with 150 mM NaCl.

Bio-mCherry-MTLS was assembled by PCR amplification from mCherry with an N-terminal biotinylation tag (the peptide MASGLNDIFEAQKIEWHEGGG, which serves as a substrate for the bacterial biotin-protein ligase BirA [61]), LZ and MACF43. Bio-mCherry-MTLS was cloned into pET28a vector linearized with EcoRI and SalI restriction enzymes. Protein purification was performed from BL21 DE3 cells. Expression was induced at OD 0.6 with 1 mM IPTG and continued overnight at 20°C. After induction, bacteria were harvested by centrifugation. The supernatant was discarded and bacteria were resuspended in 5 ml/gram lysis buffer (50 mM Tris-HCl, pH 7.4, 300 mM NaCl, 1 mM dithiolthreitol (DTT), 0.5% Triton-X, Complete Protease Inhibitor and 0.2 mg/ml lysozyme.) After ∼15 min incubation, cells were sonicated 5 times for 30 s with intervals of 1 min. The extract was pre-cleared by centrifugation at 20,000 x g for 45 min and the supernatant was incubated for 1 hr with Streptactin Sepharose beads (GE Healthcare) which had been washed 3 times in the lysis buffer. Next, beads were washed with a solution containing 2.5 mM D-biotin in the lysis buffer and spun down. The supernatant was then incubated with Ni-NTA beads (Roche) for 1 hr. Subsequently, the beads were again spun down and washed with the elution buffer containing 50 mM Tris-HCl pH 7.4, 150 mM NaCl, 300 mM imidazole, 1 mM DTT, 0.1% Triton. Finally, a PD-10 desalting column (GE Healthcare) and a concentrator (Vivaspin MWCO 10K, Satorius) were used to perform buffer exchange. The protein was snap-frozen with 10% glycerol.

Human ch-TOG was purified from HEK293T cells using the Strep(II)-streptactin affinity purification. ch-TOG expression construct was based on a previously described construct [62] that was a gift of S. Royle (University of Warwick, UK). Ch-TOG was cloned into a modified pEGFP-N1 vector with a StrepII tag. HEK293T cells were cultured in DMEM/F10 (1:1 ratio, Lonza, Basel, Switzerland) supplemented with 10% FCS, transfected using polyethylenimine (PEI, Polysciences) and harvested 2 days after transfection. Cells from a 15 cm dish were lysed in 500 μl of lysis buffer (50 mM HEPES, 300 mM NaCl and 0.5% Triton X-100, pH 7.4) supplemented with protease inhibitors (Roche) on ice for 15 min. The supernatant obtained from the cell lysate after centrifugation at 21,000 x g for 20 min was incubated with 40 μl of StrepTactin Sepharose beads (GE) for 45 min. The beads were washed 3 times in the lysis buffer without protease inhibitors. The protein was eluted with 40 μl of elution buffer (50 mM HEPES, 150 mM NaCl, 1 mM MgCl_2_, 1 mM EGTA, 1 mM dithiothreitol (DTT), 2.5 mM d-Desthiobiotin and 0.05% Triton X-100, pH 7.4). Purified proteins were snap-frozen and stored at −80°C.

Kinesin-1-GFP was purified as described previously in [57]. To generate the construct, amino acids 1-421 of the D.melanogaster kinesin heavy chain were inserted in a pET28a expression vector between the *NcoI* and EcoRI sites and GFP was inserted between the EcoRI and *XhoI* sites. The construct was transformed in the BL21DE3 bacterial strain. To express the construct, the culture was grown until OD0.6. Expression was induced with 1 mM of IPTG and cells were grown for 0.5 h at 37°C and 3.5 h at 20°C. Cells were then pelleted by centrifugation and resuspended in resuspension buffer (20 mM PIPES, 150 mM NaCl, 4 mM MgSO_4_, pH 7.0) with lysozyme and protease inhibitor cocktail (Roche). Subsequently, cells were lysed through 5 times of 30 s sonication. The soluble fraction was obtained by 40 min centrifugation at 20,000 x g and incubated with Ni-NTA beads (Roche) for 1 h at 4°C. Recombinant protein was eluted in elution buffer (80 mM PIPES, 4 mM MgSO_4_, 300 mM imidazole, 50 μM ATP, pH 7.0). The supernatant was first concentrated to 0.5 mL and buffer exchanged through gel filtration on a superdex-75 column (GE Healthcare, Superdex 75 10/300). Fractions containing kinesin-1-GFP were collected and stored at −80°C in 10% glycerol after snap-freezing in liquid nitrogen.

#### *In vitro* reconstitution assays

*In vitro* assays with dynamic MTs were performed as described previously [63]. Double cycled GMPCPP-stabilized MT seeds were prepared by incubating the tubulin mix containing 70% unlabeled porcine brain tubulin (Cytoskeleton), 18% biotin-tubulin (Cytoskeleton) and 12% rhodamine-tubulin (Cytoskeleton) at a total tubulin concentration of 20 μM with 1 mM GMPCPP (Jena Biosciences) at 37°C for 30 min. MTs were pelleted by centrifugation in an Airfuge for 5 min and then depolymerized on ice for 20 min. After this step, a second round of polymerization at 37°C with 1mM GMPCPP was performed. MT seeds were pelleted as above and diluted 10-fold in MRB80 buffer (80 mM piperazine-N,N[prime]-bis(2-ethanesulfonic acid, pH 6.8, supplemented with 4 mM MgCl_2_, and 1 mM EGTA) containing 10% glycerol, snap frozen in liquid nitrogen and stored at −80°C.

Flow chambers were assembled by sticking plasma-cleaned glass coverslips onto microscopic slides with a double-sided tape. The chambers were treated with 0.2 mg/mL of PLL-PEG-biotin (Surface Solutions, Switzerland) in MRB80. After washing with the assay buffer MRB80, the chambers were incubated with 1 mg/mL NeutrAvidin (Invitrogen). Then 2-3 μL of MTs seeds were diluted in 80 μL of MRB80 containing 127 mM glucose and attached to the biotin-NeutrAvidin links. The reaction mixtures supplemented with 20 μM of tubulin, 50 mM KCl, 0.1% Methyl cellulose, 0.5 mg/mL k-casein, 1 mM GTP, an oxygen scavenging system (200 μg/mL catalase, 400 μg/mL glucose oxidase, 4mM DTT), and 100 nM SiR-Tubulin (a fluorescent probe based on silicon-rhodamine and the MT-binding drug Docetaxel) (Cytoskeleton) were added to the flow chamber after centrifugation in an Airfuge for 5 min at 119,000 x g. In the experiments with GUVs, the vesicles were added after centrifugation. The flow chamber was sealed with vacuum grease, and dynamic MTs with and without GUVs were imaged at 30°C using Total Internal Reflection Fluorescence (TIRF) microscopy.

#### TIRF microscopy

TIRF microscopy was performed on an inverted microscope Nikon Eclipse Ti-E (Nikon) with a perfect focus system. The setup was equipped with a Nikon CFI Apo TIRF 100x1.49 N.A oil objective, Photometrics Evolve 512 EMCCD (Roper Scientific) or CoolSNAP HQ2 CCD camera (Roper Scientific) and controlled with Metamorph 7.7.5 software. The final magnification was 0.063 μm/pixel, which includes the 2.5x magnification introduced by an additional lens (VM lens C-2.5x, Nikon). The temperature was controlled by a stage top incubator INUBG2E-ZILCS (Tokai Hit). The microscope was equipped with TIRF-E motorized TIRF illuminator modified by Roper Scientific France/PICT-IBiSa, Institut Curie. For excitation we used 491 nm 100 mW Stradus (Vortan), 561 nm 100 mW Jive (Cobolt) and 642 nm 110 mW Stradus (Vortran) lasers. We used an ET-GFP 49002 filter set (Chroma) for imaging proteins tagged with GFP and NBD-PC lipid, an ET-mCherry 49008 filter set (Chroma) for imaging X-Rhodamine labeled tubulin, Rh-PE lipid or mCherry-EB3 and an ET-405/488/561/647 for imaging SiR-tubulin labeled MTs or Hilyte 647 porcine brain tubulin. We used sequential acquisition for triple-color imaging experiments.

#### Spinning disk microscopy

Spinning disk confocal microscopy was performed on a Nikon Eclipse Ti microscope equipped with a perfect focus system (Nikon), a spinning disk-based confocal scanner unit (CSU-X1-A1, Yokogawa, Japan), an Evolve 512 EMCCD camera (Roper Scientific, Trenton, NJ) attached to a 2.0X intermediate lens (Edmund Optics, Barrington, NJ), a super high pressure mercury lamp (C-SHG1, Nikon), a Roper scientific custom-ordered illuminator (Nikon, MEY10021) including 405 nm (100 mW, Vortran), 491 nm (100 mW, Cobolt), 561 nm (100 mW, Cobolt) and 647 nm (100 mW, Cobolt) excitation lasers, a set of BFP, GFP, RFP and FarRed emission filters (Chroma, Bellows Falls, VT) and a motorized stage MS-2000-XYZ with Piezo Top Plate (ASI). The microscope setup was controlled by MetaMorph 7.7.5 software. Images were acquired using Plan Fluor Apo VC 60x NA 1.4 oil objective. The 3D image reconstruction was carried out using Huygens Professional version 18.04 (Scientific Volume Imaging, the Netherlands). The temperature was controlled by a stage top incubator INUBG2E-ZILCS (Tokai Hit).

#### Stimulated emission depletion microscopy

Gated stimulated emission depletion (STED) microscopy was performed with Leica TCS SP8 STED 3X microscope driven by LAS X controlling software and using HC PL APO 100x/1.4 oil STED WHITE objective, 561 nm white laser for excitation and 775 nm pulsed lased for depletion. Depletion laser power was equal to 90% of maximum power and an internal Leica PMT detector was used. The size of the membrane tubes was estimated manually using Fiji software [64].

#### Quantification of GFP intensity along MTs

To build the average distribution of His-GFP-MTLS intensity at the MT tip (Figures S1B and S1D), we generated mean intensity profiles of 6 pixel (400 nm) thick lines with a length 2-3 μm along MTs, with the middle point positioned approximately at the MT tip [65, 66]. Previously, we extracted the fluorescence profile along a MT and fitted it with the error function, which determined MT tip position [66]. Since the profile of His-GFP-MTLS intensity had a shape of a comet, fitting it to the error function was not possible (Figure S1E). In order to use the error function to determine the position of MT tip, the maximum intensity from the His-GFP-MTLS protein fluorescence profile was assigned to all preceding points along the MT (Figure S1E) [67]. Then as described previously [65, 66], the intensity profiles *I(x)* were fitted with the error function shifted in *x* as:

(Equation 1) $$I\left(x\right)=I_{BG}+\frac{1}{2}I_{AMP}\left(1+erf\left(\frac{x-x_{c}}{\sqrt{2}\alpha }\right)\right)$$

where *I*_*BG*_ corresponds to the intensity of background, *I*_*AMP*_ the amplitude of the fluorescent signal, *x*_*c*_ is the position of the MT tip and α the degree of tip tapering convolved with the microscope’s point spread function. Each profile was shifted by its *x*_*c*_ value and background was subtracted, the 0 distance was defined as the position of the MT tip. To generate the binding curve of the His-GFP-MTLS protein (Figure S1C), profile intensities were measured at different EB3 concentrations on a single day with the same microscopy settings [68]. To estimate the mean His-GFP-MTLS intensity along the MTs (Figure 2B), we generated mean intensity profiles of 6 pixel (400 nm) thick lines along MTs.

#### Analysis of the membrane network shape

To analyze the shape of a tubular networks, we first generated single images that were maximum intensity projections of time lapse videos using Fiji (Figure S2B) [64]. A custom MATLAB script was employed to segment the network using a supervised segmentation pipeline. Segmented images (Figure S2B) were used to measure the perimeter and the area using built-in MATLAB functions from the image processing toolbox. Sphericity was calculated by dividing the total area by the squared value of the perimeter [69]. The sphericity was normalized as:

(Equation 2) $$Normalized\;sphericity=\frac{Sphericity-MIN\;\left(Sphericity\right)}{MAX\left(Sphericity\right)-MIN\left(Sphericity\right)}$$

where *max(sphericity)* and *min(sphericity)* were defined as the maximal and minimal value of the experimentally measured sphericity, respectively. Original segmented images were converted into nodes and branches (skeletonization) using built-in MATLAB functions. Skeleton representations of the networks were used to measure the total length of membrane tubes as the sum of the lengths of all detected branches (Figure S2B).

#### Analysis of thermal membrane fluctuations

Analysis of thermal fluctuations was performed as described previously [37, 70, 71]. GUVs were visualized using a phase contrast microscope (Nikon TE2000) with a 60x NA 1.4 oil objective. Fluctuations of the vesicle radius were measured at the equatorial plane by videomicroscopy. We acquired 1000 time lapse images of fluctuating GUVs. Each vesicle profile was digitalized and the GUV-equatorial fluctuations, namely *δR(x,t) = R*_*0*_*-r*_*e*_*(x,t)* were measured as local deviations of the equatorial radius *r*_*e*_ at each point of the GUV contour from an average value, *R*_*0*_, where *R*_*0*_ is calculated with respect to the center of mass of the GUV profile. The equatorial fluctuations were expanded in a series of discrete Fourier modes *n* as *δR(x,t)/R*_*0*_ *= Σ*_*q*_(*a*_*q*_*sin*(*qx*)+*b*_*q*_*cos*(*qx*)), where *a*_*q*_ and *b*_*q*_ are the Fourier coefficients and *q* = *n/R*_*0*_ (with n = 2,3,…50). The series were truncated at n = 50.

We estimated the spectrum of the membrane fluctuations in the equatorial plane *P*_*eq*_ (in units of lengthˆ^3^) by calculating time-averages of quadratic fluctuation amplitudes: *P*_*eq*_ = *A*^∗^(〈|*c*_*n*_|^2^〉-〈|*c*_n_|〉^2^), where *c*^*2*^_n_ = *a*^*2*^_n_+*b*^*2*^_n_ and *A =* π〈*R*_*0*_〉^*3*^*/2* (Figure S3C, left). Since the amplitudes of the fluctuations estimated from experiments depended on the GUV mean radius 〈*R*_*0*_〉, non-dimensional averaged curves were obtained after dividing each individual spectrum by *A =* π〈*R*_*0*_〉^*3*^*/2* (Figures 2E and S3C, right) [37, 70]. The estimated amplitude of the Fourier modes included errors due to the finite spatial resolution of the images (pixelization). This noise introduces in the fluctuations a systematic component, which could become dominant at high fluctuation modes [70, 72]. Consequently, the experimentally measured amplitudes were corrected by subtracting the noise as *P*_*eq*_ (corrected) = *P*_*eq*_ − [(π〈*R*_*0*_〉^*3*^*/2*)^∗^var(*c*_*n*_)], where var(*c*_*n*_) represents the variance of *c*_*n*_ as explained in [70]. A detailed derivation of the formulas for error propagation is provided in the Appendix C of [70]. In addition, we quantified the minimal fluctuation that we can estimate by measuring the fluctuation spectrum of a fixed object in the focal plane (Figure 2E). As a model for a fixed object, we used a transparent circular disc of radius 10 μm, printed in a Quartz photomask coated with chrome (Toppan). Every fluctuation amplitude below the estimated resolution limit was discarded during fitting. A correction to account for to the finite integration time of the camera [70, 73] was also included in the analysis.

Mechanical parameters of the GUVs were obtained by fitting the experimental mode amplitudes to the theoretical spectrum for a planar membrane [37, 70]. Detection of the vesicle contours, post-processing and fitting procedures were carried out using a custom MATLAB script as described previously [37].

#### Analysis of MT and membrane dynamics

To analyze the dynamics of MTs and membrane tubes, kymographs were generated using Fiji (KymoResliceWide plugin, https://github.com/ekatrukha/KymoResliceWide). MT growth rate and the speed of extension of membrane tubes were determined manually from kymographs. To estimate the probability of tubular deformations and detachment events, we selected MTs growing beyond the visible contour of the GUV (Figures 3G, S4F, and S4G) and divided the number of observed tubular membrane deformations by the total number of MT growth events. To estimate the probability that a deformation stopped because the membrane tip detached from the MT tip, we divided the total number of detachment events by the total number of successful membrane deformations (Figures 3G and 3I).

To determine the spreading speed of a membrane tube, time lapse images of GUVs interacting with MTs were recorded at 3 s intervals with 200 ms exposure time for 5 or 10 min. Intensity profiles of membrane tubes labeled with Rh-PE were collected at different time points by averaging across 6-pixel (400 nm) wide lines. The position of a tube end was determined by fitting every profile with the error function using a custom written MATLAB script [65] (Figure S5A). Membrane tube spreading speed was calculated by differentiating the vector of the tube end position over time.

#### Reconstitution of Qdot transport with MT tips

Digoxigenin (DIG)-labeled tubulin was prepared as described previously [74]. All other tubulin products were purchased from Cytoskeleton. Chambers were prepared and imaged as described previously [41]. In brief, silanized slides and coverslips were assembled into chambers using double-sided tape and functionalized with anti-DIG IgG (Roche), then passivated with 1% Tween-20. Double-cycled, DIG-labeled GMPCPP-stabilized seeds were introduced, followed by the reaction mix containing 80 mM K-Pipes pH 6.9, 50 mM KCl, 1 mM EGTA, 4 mM MgCl_2_, 1 mM GTP, 0.1% methylcellulose, 1 mg/ml κ-casein, 4 mM DTT, 0.2 mg/ml catalase, 0.4 mg/ml glucose oxidase, 20 mM glucose, 20 μM tubulin (5% labeled with HiLyte-488) and 30 pM Qdot705-streptavidin (Thermo Fischer) with or without the addition of 15 nM bio-mCherry-MTLS and 100 nM mCherry-EB3.

Images were acquired using Nikon Ti-E microscope (Nikon, Japan) with the perfect focus system (Nikon) equipped with a Plan Apo 100X 1.45 NA TIRF oil-immersion objective (Nikon), iLas^2^ ring TIRF module (Roper Scientific) and a Evolve 512 EMCCD camera (Roper Scientific, Germany). The sample was illuminated with 488 nm (150 mW for HiLyte488-tubulin), 561 nm (100 mW for mCherry-tagged proteins) and 642 nm (110 mW, for Qdot-705) lasers through a quad-band filter set containing a ZT405/488/561/640rpc dichroic mirror and a ZET405/488/561/640 m emission filter (Chroma). Images were acquired sequentially with MetaMorph 7.8 software (Molecular Devices, San Jose, CA). The final resolution was 0.16 μm/pixel. The objective was heated to 34°C by a custom-made collar coupled with a thermostat, resulting in the flow chamber being heated to 30°C.

#### Optical trap experiments

1 μm glass beads functionalized with carboxy groups (Bangs Laboratories) were conjugated with PLL-PEG (Poly-L-lysine (20 kDa) grafted with polyethyleneglycole (2 kDa), SuSoS AG, Switzerland) supplemented with 10% v/v of PLL-PEG-biotin as described [41]. The bead surface was then saturated with Neutravidin and then Bio-mCherry-MTLS. Flow chambers were prepared as described above. Reaction mix contained 80 mM K-Pipes pH 6.9, 50 mM KCl, 1 mM EGTA, 4 mM MgCl_2_, 1mM GTP, 1 mg/ml κ-casein, 4 mM DTT, 0.2 mg/ml catalase, 0.4 mg/ml glucose oxidase, 20 mM glucose and either 25 μM unlabelled tubulin with addition of 100 nM mCherry-EB3, or 10 μM unlabelled tubulin in the absence of EB3. Experiments were carried out at 25°C.

DIC microscopy and optical trapping were performed as described previously [41]. Measurements were performed at nominal trap power of 0.4W which resulted in a typical stiffness of 0.03-0.05 pN/nm. The quadrant photo detector (QPD) signal was recorded at 10 kHz. For experiments measuring forces in the direction of the MT growth, which were expected to be in the sub-pN range, the 0.2W trap (the lowest setting for our instrument) was additionally softened down to 4-6 ·10^−3^ pN/nm using a circular polarizing filter placed in the wave path of the trapping laser. The QPD signal was recorded at 10 kHz and down-sampled to 1 kHz for analysis.

#### Single molecule measurements

His-GFP-MTLS single molecule binding events to MTs were recorded using TIRF microscopy (Figure S5C) as described previously [75]. Briefly, the assay was performed at 0.12 nM concentration of His-GFP-MTLS with high speed acquisition (50 ms/frame). 200 nM mCherry-EB3 was added as a tracer to detect MT plus ends. The number and dwell times of the binding events were extracted from kymographs that were generated using Fiji (KymoResliceWide plugin). The distributions of the dwell times were fitted with an exponential function, giving the dissociation rates from MT lattice and from MT tips (Figures S5D and S5E). The binding constant rate was determined by measuring the number of single molecule binding events per MT lattice length and assuming a length of 200 nm for the MT tip (Figure S5F).

The diffusion coefficient of His-GFP-MTLS on MT lattice *D*_*MT*_ (Table S1, parameter 14) was derived from the analysis of the mean square displacement (MSD) of single molecule spots diffusing along MTs [75] (Figures S5C and S5G). Coordinates of diffusing spots were obtained and linked across frames using the Fiji plugin TrackMate [76]. MSD analysis was performed using MSDanalyzer MATLAB routine [77]. 25% of each MSD curve excluding zero was used for fitting. To compute the mean *D*_*MT*_ we only considered values where the coefficient of determination *R*^*2*^ of the fitting was above 0.8 (n = 95)(Figure S5G).

#### Fluorescence correlation spectroscopy

We used z-scan Fluorescence Correlation Spectroscopy (z-FCS) to measure the diffusion coefficient of His-GFP-MTLS bound to GUVs [78]. z-FCS measurements were performed with a Leica SP8 STED 3X microscope driven by LAS X and SymPhoTime (PicoQuant) software using a HC PLAPO 63x 1.2 NA water immersion objective. The sample was excited at 488 nm and time correlation was calculated at the time frequency of 600 kHz.

Confocal imaging was used to localize the top of the GUVs, starting from which a fast scan along the z axis was performed. The position of the focus in z was set at 1 μm below or above of the plane with the maximum intensity. Afterward, autocorrelation functions *G(t)* were measured at different positions along the z axis in 0.2 μm steps. Three traces of 10 s duration were acquired at each position. The autocorrelation functions were fitted with the model considering one single species diffusing in two dimensions described previously [79]:

(Equation 3) $$G\left(t\right)=\frac{1}{N_{p}}{\left(1+\frac{t}{t_{D}}\right)}^{-1}$$

where *N*_*p*_ is the average number of particles in the confocal volume, _*tD*_ is the characteristic diffusion time and *t* is the lag time. The obtained average number of particles and diffusion times was plotted against the focus position *z* and fitted with the equations derived previously [80]:

(Equation 4) $$t_{D}=\frac{w_{0}^{2}}{4D_{m}}{\left(1+\frac{\lambda_{0}^{2}\Delta z^{2}t}{\pi^{2}n^{2}w_{0}^{4}}\right)}^{-1}$$

(Equation 5) $$N_{P}=\pi cw_{0}^{2}{\left(1+\frac{\lambda_{0}^{2}\Delta z^{2}t}{\pi^{2}n^{2}w_{0}^{4}}\right)}^{-1}$$

where *w*_*0*_ (Table S1, parameter 18) is the radius of the beam in the focal plane, *D*_*m*_ (Table S1, parameter 16) is the diffusion coefficient of the protein bound to membrane, _*c*_ is the average concentration in the illuminated area, *n* is the refractive index of the medium and λ is the wavelength of the excitation light.

We used FCS to estimate the probability of finding His-GFP-MTLS bound to EB3-bound MT tip as a function of the distance from the MT tip (Figure 6C). We reconstituted MT plus-end tracking of the His-GFP-MTLS (15 nM) and EB3 (200 nM) as before, but in the absence of GUVs. When we performed FCS experiments, we observed peaks in the fluorescence intensity trace (Figure 6B). The peaks represented His-GFP-MTLS-labeled MT ends [30, 81]. From the intensity values next to the intensity peak we calculated the autocorrelation function as described above. The autocorrelation function *G*(*t*) was fitted using a triplet-state model for one fluorescent species [82] as follows:

(Equation 6) $$G\left(t\right)=\frac{1}{N_{p}}\left[1-T+Te^{\left(-\frac{t}{t_{T}}\right)}\right]{\left(1+\frac{t}{t_{D}}\right)}^{-1}{\left(1+\frac{t}{t_{D}S^{2}}\right)}^{-1/2}$$

where *T* is the triplet decay fraction, *t*_*T*_ is the lifetime of the triplet state, *t*_*D*_ is the diffusion time of the fluorescent species and *S* is the length-to-diameter ratio of the focal volume set to 4. We then estimated the mean of the maximum numbers of His-GFP-MTLS molecules at the MT tip *< N*_*p-tip*_ > as:

(Equation 7) $$\langle N_{p-tip}\rangle =\frac{N_{p}\langle I_{peak}\rangle }{\langle I_{solution}\rangle }$$

where < *I*_*peak*_ > is the mean peak intensity measured during 1 s (Figure 6B), and < *I*_*solution*_ > is the averaged intensity measured after the peak (Figure 6B). It was assumed that the intensity ratio was proportional to the ratio of the numbers of His-GFP-MTLS molecules at the MT tip and in solution. Considering that MT growth rate is ∼2.8 μm/min, we estimated that the maximum intensity recorded in 1 s corresponded to ∼46 nm of the MT tip (∼6 tubulin dimers). Since EB3 binds to MTs between protofilaments at the interface between four tubulin dimers but not at the seam [49] and assuming that EB3 and MTLS interact at a ratio of 1:1, we considered that within 6 dimer lengths of a 13 protofilament MT there were ∼60 potential binding sites for His-GFP-MTLS. Considering that at 200 nM EB3 all EB3 binding sites could be occupied, we estimated that the maximum probability of finding His-GFP-MTLS bound to the EB3-bound MT tip was < *N*_*p-tip*_ > /60 = 28/60 = 0.46 (see Table S1, parameter 13). We then estimated the probability of His-GFP-MTLS binding along the MT length as the product of the maximum probability calculated above using normalized average His-GFP-MTLS intensity profiles measured as in Figure S1B.

#### Estimation of His-GFP-MTLS surface density

To estimate the surface density of His-GFP-MTLS molecules bound to the GUV surface in contact with MTs (*ρ*_*MTLS-MT*_) (Table S1, parameter 21) we used FCS and fluorescence intensity analysis. As we described above, we measured by FCS the average number of free His-GFP-MTLS molecules bound to the surface of the GUV in the confocal volume *N*_*p*_ and the radius of the beam in the focal plane *w*_*0*_. Then we estimated the density of free His-GFP-MTLS molecules bound to the surface of the GUV (Table S1, parameter 20) as the ratio between the average number of His-GFP-MTLS molecules and the area of the beam in the focal plane: *ρ*_*MTLS-MT*_ *= N*_*p*_ /*w*_*0*_^*2*^. Finally, the density of the His-GFP-MTLS molecules bound to the GUV surface in contact with MTs was determined as: *ρ*_*MTLS-MT =*_ *ρ*_*MTLS*_*^∗^(I*_*GUV-MTs contact*_ */I*_*GUVS*_*)* where *I*_*GUV-MTs contact*_ is the mean fluorescent intensity of the free His-GFP-MTLS bound to the surface of the GUV in contact with MTs *I*_*GUV-MTs*_ and *I*_*GUVS*_ is the fluorescent intensity of the free His-GFP-MTLS molecules bound to the surface of the GUV (Figure 2B).

#### Physical modeling

##### Membrane sliding along MT lattice

To describe membrane sliding along MT lattice, a minimal one dimensional (1D) theoretical model was adapted from a previous publication [39]. We considered that the binding-unbinding reaction of His-GFP-MTLS to MT takes place in the close vicinity of the tip of a membrane deformation. The width of the reaction zone _*d*_ in the 1D model was fixed to the length of one tubulin dimer, *d* = 8 nm. Moreover, we considered a reaction-dominated regime because of the high His-GFP-MTLS concentration at the GUV-MT interface (Figures 2A and 2B). For simplicity, we first considered only the tip of the tube that extended for a distance *L*. We assumed that the tip moves in a biased-random walk, stepping from one binding site to the next with the forward rate *k*_*on-m*_ and backward rate *k*_*off-m*_. The unbinding rate depends on the force barrier *F*_*m*_*(t)* to pull a membrane tube as described previously [23].

(Equation 8) $$k_{off-m}\left(F_{m}\right)=k_{off-m}*exp\left[\frac{F_{m}\left(t\right)\xi }{k_{B}T}\frac{1}{n_{p}}\right]$$

where *F*_*m*_(*t*) *= 2π*(*2σ*(*t*)κ)^1/2^ [38], κ is the bending modulus and σ(*t*) is the lateral tension of the membrane. The parameter ξ (Table S1, parameter 6) is the characteristic length of the potential barrier between bound and unbound states, *T* is the temperature, *k*_*B*_ the Boltzmann’s constant and *n*_*p*_ (Table S1, parameter 7) is the number of proteins bound at the tip. At the tip, the load is equally distributed between all the bonds due to the parallel organization (Figure 5A). The lateral tension of a flaccid membrane with the initial tension *σ*_*0*_, increased during tube elongation as described in [36]:

(Equation 9) $$\sigma \left(t\right)=\sigma_{0}*exp\left[\frac{8\pi \kappa }{k_{B}T}\alpha \right]$$

where *α = (A-A*_*0*_*)*/*A*_*0*_ is the relative area change of the GUV during tube elongation, *A*_*0*_ *= 4πR*^*2*^ is the initial surface area of the GUV, *R* the GUV radius and *A = 2πr*_*0*_*L*(*t*)+*4πR*^*2*^ is the total surface area of the GUV after a tube was pulled; *r*_*0*_ is the curvature radius of the tether and *L(t)* the tether length. Based on the considerations above, we described the dynamics of a sliding membrane with a one-point one-step master Equation [44, 83]. The probability that the tip of a sliding membrane ends up at the site *n* on the MT lattice at the time *t+Δt* starting from the time *t* is the sum over all of the individual paths available to the system with the condition that the final state is fixed to one and can be written as:

(Equation 10) $$\begin{array}{cccc} & \text{Stepping forward} & \text{Stepping backward} & \text{Stay at site n}\\ p\left(n,t+\Delta t\right)= & k_{on-m}\Delta tp\left(n-1,t\right)+ & k_{off-m}\left(F\right)\Delta tp\left(n+1,t\right)+ & \left(1-k_{on-m}\Delta t-k_{off-m}\left(F\right)\Delta t\right)p\left(n,t\right) \end{array}$$

We transformed this equation into the more familiar continuum form as the function of the position *x* along the MT lattice, where the probability *p(x,t)* for finding the tip of the deformation at position *x* is equal to *p(n,t)* when *x = nd,* as was done in [44]. This led to a result that represents a biased diffusion equation for the front of the sliding membrane of the deformation tip:

(Equation 11) $$\frac{\partial p}{\partial t}=-V\frac{\partial p}{\partial x}+D\frac{\partial^{2}}{\partial x^{2}}$$

where

(Equation 12) $$V=d\left[k_{on-m}-k_{off-m}\left(F_{m}\right)\right]\;$$

and

(Equation 13) $$D=\frac{d^{2}}{2}\left[k_{on-m}+k_{off-m}\left(F_{m}\right)\right]$$

*V* is the average speed of the membrane tip and *D* is the variance of the tip position due to the stochastic nature of the process. As can be seen from (Equation 8), (Equation 12) depends on *F*_*m*_*(t)* or, in the context of tip’s probability function *p*, ultimately on *x*. To generate the dependence of speed for different time points of spreading (depicted in the Figures 5B and S5B), we used iterative estimation of *x*(*t*) over time series with step *Δt*:

(Equation 12a) $$x_{n+1}\left(\left(n+1\right)\cdot \Delta t\right)=x_{n}\left(n\Delta t\right)+V_{n}\left(x_{n}\right)\Delta t$$

with the initial condition *x*(0) = 0, which at the end provided values of *V*_*n*_ for each time point *n Δt.* We chose the value of *Δt* = 0.5 s, and the variation in this parameter in the range of 0.1-5 s did not affect the final result. Curves in Figure 5B are averages of multiple solutions of Equation 12a with different values of the physical parameters of the GUVs.

#### Parametrization of the model

We assumed that the affinity of the membrane for the MT is determined by the binding properties of the His-GFP-MTLS to MT. The value of the *k*_*off-m*_ *=* 1.7 s^-1^ was defined as the inverse of the dwell time of the His-GFP-MTLS on the MT lattice, obtained from the analysis of the His-GFP-MTLS single molecule binding events (Figures S5C–S5F). We generated the curves in Figure 5B with three different values of *k*_*on-m*_ = (8,16,26) s^-1^. In our model, only *k*_*off-m*_ was modified by the force required to pull a membrane tube as described by Equations 8 and 9.

The mechanical parameters of the membrane were obtained from the analysis of thermal fluctuations of the GUVs (Figures 2E and 2F). The precise value of the characteristic length of the potential barrier ξ between bound and unbound states is unknown for our system. However, the values reported in different systems are in the range between 1-2 nm [84, 85]. For modeling, we fixed ξ to 1 nm.

The radius of curvature of the deformation *r*_*0*_ defines the number of His-GFP-MTLS molecules bound to the tip. In the 1D model, *r*_*0*_ was represented by a fixed length *L = dn*_*p*_ in the range between 8 and 32 nm [23]). For modeling, the number of His-GFP-MTLS molecules bound to the tip was randomly selected between 1 and 3.

The curves in Figure 5B are averages of 100 individual traces. For each trace, the lateral tension and the bending modulus of the simulated membrane were randomly sampled from a normal distribution with the mean and variance obtained from the analysis of the thermal fluctuations of the GUVs (Figures 2E and 2F). The radius *R* was sampled from a uniformly randomly distributed values ranging between 5 and 15 μm.

#### Stochastic simulations

##### Membrane sliding along MT lattice

To simulate the dynamics of membrane tubes sliding along MTs, we have adapted the previously developed model for motors pulling fluid membranes [23]. His-GFP-MTLS molecules were represented by particles. MTs were represented by a discrete one-dimensional (single protofilament) lattices with *M* grid cells numbered from left to right (from the seed to plus end), and the membrane was described by a one-dimensional discrete lattice with *N* grid cells with *N* < *M,* also numbered from left to right (Figure 5D). The condition *N* < *M* guaranteed that the substrate for the adhesion was always available. Lattice spacing was fixed to the size of a tubulin dimer *d* = 8 nm. Individual MT cells were considered attachment sites with the capacity of one molecule and individual membrane sites were reservoirs of His-GFP-MTLS molecules. The number of His-GFP-MTLS molecules in a given cell at the MT grid represented the MT occupation number *n*_*MT*_. Similarly, the number of His-GFP-MTLS molecules occupying each site on the membrane was denoted as the membrane occupation number *n*_*m*_. Along the MT, His-GFP-MTLS molecule attachment sites were either empty (*n*_*MT*_ = 0) or occupied (*n*_*MT*_ = 1). We restricted the number of His-GFP-MTLS molecules attached to the MT at a given site to 1. However, along the membrane, each cell in the grid was unoccupied or occupied by several His-GFP-MTLS molecules.

The specified transitions of the His-GFP-MTLS molecules attached to a MT were:

1 Detachment from the MT with a rate *k*_*off-m*_*.* If the transition occurred at the tip of the deformation, the tube retracted to the next site containing a bound His-GFP-MTLS molecule.
2 Diffusion to the left or to the right with a rate *k*_*D-MT*_, if the site was empty.
3 Diffusion to the right from position *N* was not allowed.
4 Diffusion to the right from position 1 increased the number of proteins in the deformation by one.

The putative transitions of the His-GFP-MTLS molecules detached from a MT were:

5 Attachment to the MT with a rate *k*_*on-m*_ if the site on the MT was unoccupied. If the transition happened at the tip, the deformation was extended by one site.
6 Diffusion to the left or to the right with a rate *k*_*D-M*_ (Table S1, parameter 17) if the site was empty.

In the particular case of a membrane spreading along a MT lattice, the on and off rates along the deformation remained constant; however, at the tip of the deformation, the dissociation rate depended on the force barrier as explained above (see Equation 8).

##### Membrane spreading at the tips of growing MTs

The stochastic simulations describing the behavior of spreading membrane tubes in the presence of dynamic MTs included some additional features. As before, the MT was represented by a discrete 1D lattice, but with *N* grid cells numbered from left to right, and the membrane was described by a discrete 1D lattice with *M* grid cells (Figure 6A). A feature of the simulation in the presence of growing MTs was the stochastic dynamics of the MTs. In the reaction scheme for MTs that was used in the simulations, MTs could be either in a growth phase or in a shrinkage phase.

For a MT in the growth phase, the possible events were:

1 Association of a tubulin dimer at position *N*: The length of the MT was increased by one dimer. The length of the tube was unchanged.
2 Dissociation of a tubulin dimer at position *N*: The length of the MT was decreased by one dimer. If the membrane was spreading up to the tip (*M* = *N*), it would also shrink to the next site containing a bound His-GFP-MTLS molecule.
3 Catastrophe: A change in the state from the growth phase to the shrinkage phase.
4 Rescue: A change in the state from the shrinkage phase to the growth phase.

The specified transitions of the His-GFP-MTLS molecules attached and detached from a MT were as before with the following special rule for His-GFP-MTLS molecules detached from a MT:

5 Attachment to the MT with a rate *k*_*on-m*_ if the site on the MT was unoccupied. If the transition happened at the tip of the membrane, the deformation was extended by one site. If the membrane was spreading up to the tip (*M* = *N*) the transition was not allowed, because in our experiments, the membrane was never observed to spread beyond the MT tip.

As before, the dissociation rate was constant along the MT, but in the vicinity of the deformation tip, the rate depended on the force barrier. The association rate was regulated in space and time by the lack or presence of EB3.

##### Boundary between the tube and the vesicle

Position 1 in the grid characterized the connection of the deformation with the GUV [23] and constituted the only source of His-GFP-MTLS molecules entering the membrane tether. We assumed that there is no influx of free His-GFP-MTLS molecules from solution. Since the linear density of His-GFP-MTLS molecules in the deformation must be constant over time, at this position the occupation numbers of the attached and detached molecules were kept constant and equal to 1 (*n*_*MT*_ = *n*_*m*_ = 1). Diffusion from site 2 to 1 was not allowed, to keep the number of molecules in the deformation constant over time. The diffusion rate of the proteins that were not attached to the MT at position 1 was different from the other sites in the grid and was defined as *k*_*D-M1*__=_ *ρ*_*MTLS*_*^∗^V*_*tube,*_ where *ρ*_*MTLS*_ is the linear density of the His-GFP-MTLS molecules in the deformation and *V*_*tube*_ is the growth speed of the tube. This condition accounted for the influx of molecules entering the deformation due to the membrane flow resulting from the tube growth.

##### Density of His-GFP-MTLS molecules

At the beginning of the simulation, the linear density of the His-GFP-MTLS molecules was estimated as described previously [23]: *ρ*_*MTLS-linear*_ *= ρ*_*MTLS-Dlinear*_*+ ρ*_*MTLS-Alinear,*_ where *ρ*_*MTLS-Dlinear*_ and *ρ*_*MTLS-Alinear*_ were the linear density of the His-GFP-MTLS molecules detached and attached to the MT respectively. The linear density were defined as*: ρ*_*MTLS-Dlinear*_ *= 2π r*_*0*_ *ρ*_*MTLS-MT*_ (*k*_*off-m*_ /(*k*_*off-m*_ + *k*_*on-m*_)) and *ρ*_*MTLS-Alinear*_ *= 2π r*_*0*_ *ρ*_*MTLS-MT*_ (*k*_*on-m*_ /(*k*_*off-m*_ + *k*_*on-m*_)), *ρ*_*MTLS-MT*_ was the surface density of His-GFP-MTLS molecules at GUVs surface in contact with MTs estimated experimentally, and *r*_*0*_ was the curvature radius of the deformation.

##### Simulation parameters in the absence of EB3

As before, the value for *k*_*off-m*_ = 1.7 s^−1^ was defined as the inverse of the dwell time of His-GFP-MTLS molecules on MT lattice, obtained from the analysis of the single molecule binding events (Figures S5C–S5F). The stochastic rate of the detachment reaction was *a*_*off-m*_ = *k*_*off-m*_^∗^*n*_*MT*_ . As explained above, the dissociation rate remained constant along the MT, but was allowed to change at the very end of a membrane deformation. The first *n*_p_ His-GFP-MTLS molecules bound to the MT at the tip of a deformation shared the force to pull a tube. The parameter *n*_p_ was determined as a value of MT occupancy *n*_*MT*_ of the last four elements on the outermost membrane tip. The parameters *σ*_*0,*_*κ* and *R* were random variables sampled from a normal distribution. The parameter ξ was fixed to 1 nm.

To determine the value of *k*_*on-m*_ we compared the results obtained from the one-step model using different values for *k*_*on-m*_ (Figures 5B and S5B). Based on the experimental data (Figure S5B), we found that *k*_*on-m*_ = 16 s^−1^ was a reasonable value to use in the stochastic simulations. This value of *k*_*on-m*_ is the attachment rate of His-GFP-MTLS to MT lattice, which is constant along the MT, and we assumed that was not affected by the tube force. In the absence of EB3, *k*_*on-m*_ had the same value along the MT shaft and at the MT tip, because His-GFP-MTLS molecules at the front of the deformation cannot distinguish between MT shaft and MT tip. The stochastic rate of the attachment transition was *a*_*on-m*_ = *k*_*on-m*_^∗^*n*_*m*_.

##### Simulation parameters in the presence of EB3

In the presence of EB3, the formation of a His-GFP-MTLS-EB3 complex imposed a spatial and temporal regulation on *k*_*on-m*_. The association rate of His-GFP-MTLS at MT tips that were not in contact with membranes was 2.5 fold higher than the on-rate of His-GFP-MTLS at the MT shaft (Figure S5F). We assumed that the kinetic constants of His-GFP-MTLS bound to the membrane displayed the same ratio, resulting in *k*_*on-m-tip*_ = 2.5 ^∗^ *k*_*on-m*_.

As mentioned above, every elemental stochastic reaction at a given position in the grid depends on the number of available molecules (occupation number). The number of EB3 molecules is high at the MT plus ends and decays toward the seed along the lattice of the MT, showing a comet-like shape. The distribution of the number of His-GFP-MTLS molecules measured from experiments showed the same pattern (Figures 1E and S1B). The stochastic rate of the attachment reaction of His-GFP-MTLS to the MT tip in the presence of EB3 can then be written as *a*_*on-m-tip*_ = *k*_*on-m-tip*_^∗^*n*_*m*_^∗^*p*_*m-MTLS*,_ where *p*_*m-MTLS*_ is the occupation probability of His-GFP-MTLS along a MT starting from the tip measured experimentally (Figure 6C). His-GFP-MTLS molecules that detached from a MT diffused along the membrane with equal probability to the left or to the right. In the discretized representation, the diffusion of the proteins along the deformation is characterized by a constant rate *k*_*D-m*_ = *D*_m_/*d*^*2*^ (ref [23]) with a stochastic rate *a*_*D-m*_ = *k*_*D-m*_^∗^*n*_*m*_ where *D*_*m*_ is the diffusion coefficient of the protein on the surface of the GUVs (Figures S5H and S5I).

Similarly, we considered symmetric diffusion of His-GFP-MTLS molecules bound to a MT, with the stochastic rate *a*_*D-MT*_ = *k*_*D-MT*_^∗^*n*_*MT*_ and *k*_*D-MT*_ = *D*_MT_/*d*^*2*^. Here, *D*_MT_ is the diffusion coefficient of

His-GFP-MTLS molecules bound to the MT. We assumed that the diffusion rates were not affected by the presence of EB3 or by membrane forces.

#### Gillespie algorithm: first-family method

The system was characterized by a rate matrix *k*_*S*_*,*_*F*_ where *S = 1,…N* indicates the sites in the 1D lattice representation and *F = 1,…11* the number of potential transitions of the His-GFP-MTLS molecules. To implement the first-family method, we considered every transition *F* as a family with *S* potential reactions in each family [46]. Every family *F* is then considered as a pseudoreaction with the stochastic rate a^F^_0_≡Σ^S^_j = 1_*k*^*F*^_*j*_^∗^*n*_*s*_, where *k*^*F*^_*j*_ is the rate of the family *F* and *n*_*s*_ *=* (*n*_*m*_ or *n*_*MT*_) is the occupation number at the position *S*. To generate the time *t* to the next reaction event and the index pair (*F,S*) that identifies the type of transition and the position, we generated *F*+1 random numbers *r*_1_,…*r*_F+1._ We used the first *F* numbers to calculate

(Equation 14) $$t_{F}=\frac{1}{a_{0}^{F}}ln\left(\frac{1}{r_{F}}\right)\left(F=1,\ldots 11\right)$$

then

(Equation 15) $$\begin{array}{c} t=min\left(t_{F}\right)\\ F=the\;index\;of\;the\;smallest\;t_{F}\; \end{array}$$

From the above, we determined the type of the next transition (detachment, attachment, diffusion, MT polymerization, MT depolymerization and MT transitions between growth and shrinkage). The position *S* of the transition in the grid was determined as:

(Equation 16) $$S=min,m\in N,\;s.t.\sum_{m=1}^{m}a_{S}^{F}>r_{F+1}a_{0}^{F}\sum_{m=1}^{m}a_{S}^{F}>r_{F+1}a_{0}^{F}$$

where *a*^*F*^_*S*_ *= k*^*F*^_*S*_^∗^*n*_*s*_ and *k*^*F*^_*S*_ is the rate of the family *F* at position *S* on the grid. All the simulations were performed in MATLAB using custom routines

#### Postprocessing of the simulation output

The output of each individual simulation was the position of the membrane tip (sliding along a MT shaft), or the position of the membrane and MT tip. We applied the following rules to sort the deformation events:

1 Events with a deformation of length shorter than 200 nm were regarded as non- deformation events with zero length.
2 Events classified as deformations (length > 200 nm) were categorized as short tubes when the tube length was the range of 200-600 nm.
3 Tubes with a length > 600 nm were considered as long deformations.
4 Only MTs that grew more than 200 nm were considered for analysis.
5 Events were classified as detachment events when the distance between the membrane tip and the MT tip was longer than 200 nm.

### Quantification and Statistical Analysis

Data analysis was performed with GraphPad, MATLAB and Python running on Jupyter Notebook. Mean values are shown in the graphs, and details of error bars and sample sizes can be found in the figure legends. To estimate the probability of tubular deformations and detachment events, we selected MTs growing beyond the visible contour of the GUV and divided the number of observed tubular membrane deformations by the total number of MT growth events. To estimate the probability that a deformation stopped because the membrane tip detached from the MT tip, we divided the total number of detachment events by the total number of successful membrane tubulation events. Kymographs were generated using Fiji (KymoResliceWide plugin, https://github.com/ekatrukha/KymoResliceWide). MT growth rate and the membrane tube extension were determined manually from kymographs.

### Data and Code Availability

All data that support the conclusions are available from the authors on request, and/or available in the manuscript itself. Data analysis was performed in MATLAB or using GraphPad Prism version 8.00 for (Mac OS X). The custom software used for data analysis and simulations in this manuscript can be found at https://github.com/RuddiRodriguez/; https://github.com/ekatrukha/KymoResliceWide. The software for the analysis of the GUV thermal fluctuations was developed M. Mell, I. López Montero and R. Rodríguez-García at the Complutense University in the group of F. Monroy and is available at https://github.com/RuddiRodriguez/programnnn.

The computer code for simulations and analysis is available in https://github.com/RuddiRodriguez/Spreading;https://github.com/RuddiRodriguez/Spreading_membrane-MT_reaction_inside_EB.

Schematic representations were generated in Adobe Illustrator with the support of ChemDraw (PerkinElmer Informatics) and Biorender (©BioRender - biorender.com).

## Supplemental Information

Document S1. Figures S1–S5 and Table S1

Document S2. Article plus Supplemental Information
