# Supplementary material for: Mechanisms of Motor-Independent Membrane Remodeling Driven by Dynamic Microtubules
Source: Curr Biol. 2020 Mar 23;30(6):972–987.e12. doi: 10.1016/j.cub.2020.01.036 (PMC7090928; doi:10.1016/j.cub.2020.01.036)
Supplement: Document S1. Figures S1–S5 and Table S1 [file mmc1.pdf]

Current Biology, Volume *30*

Supplemental Information

Mechanisms of Motor-Independent Membrane Remodeling Driven by Dynamic Microtubules

Ruddi Rodríguez-García, Vladimir A. Volkov, Chiung-Yi Chen, Eugene A. Katrukha, Natacha Olieric, Amol Aher, Ilya Grigoriev, Magdalena Preciado López, Michel O. Steinmetz, Lukas C. Kapitein, Gijsje Koenderink, Marileen Dogterom, and Anna Akhmanova

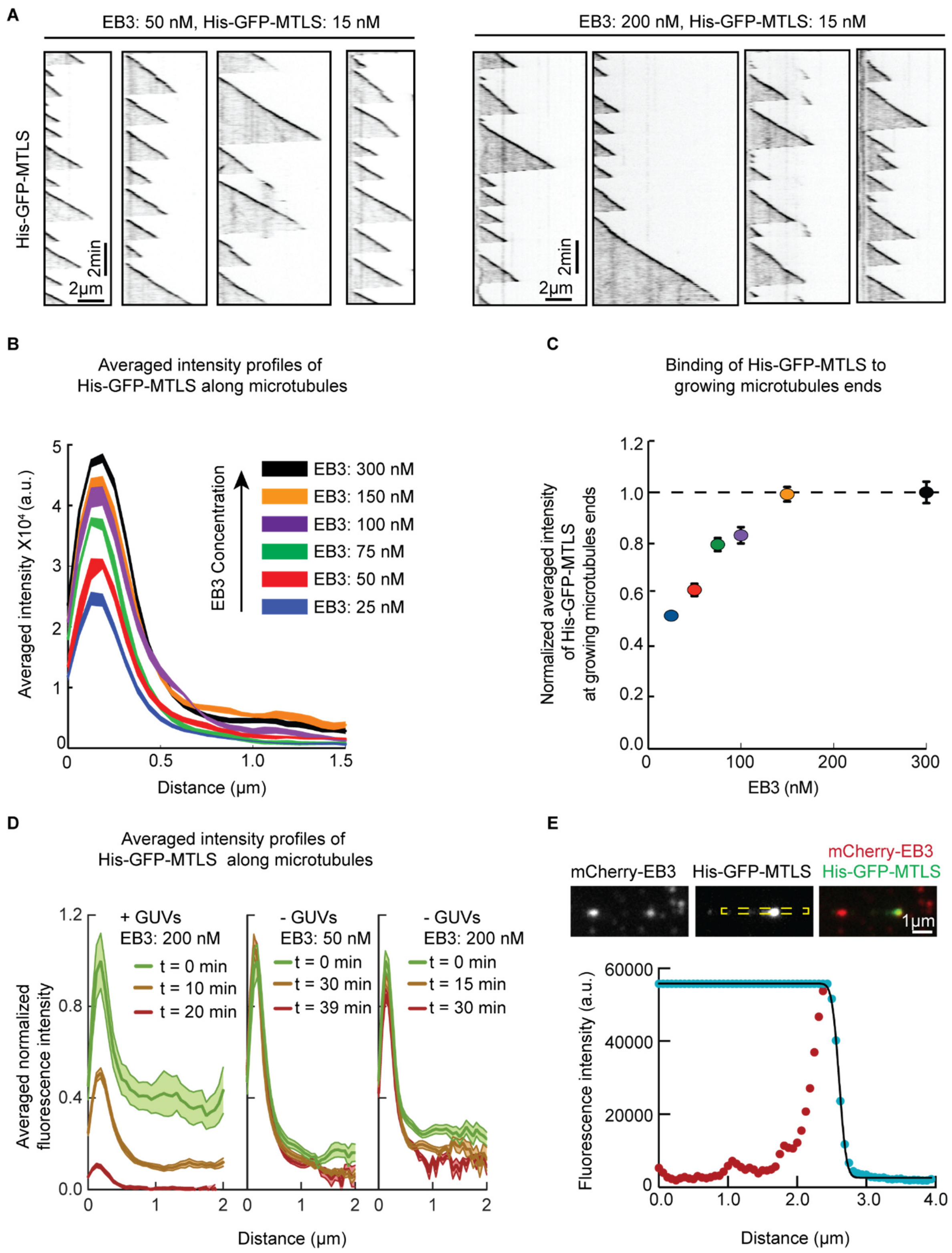

Figure S1. Analysis of the distribution of His-GFP-MTLS along MTs, Related to Figure 1.

(A) Representative kymographs illustrating the dynamics of MTs grown in presence of 15 nM His-GFP-MTLS at different concentrations of EB3.

(B) Averaged tip intensity profiles for His-GFP-MTLS in the presence of different EB3 concentrations and 20 μm of porcine tubulin. The shaded areas represent SEM. From low to high EB3 concentration, n numbers: 65, 134, 37, 75, 102, 93.

(C) Averaged maximum His-GFP-MTLS fluorescence intensities at MT plus ends as a function of EB3 concentration. Data were normalized to the average maximum intensity obtained at the highest EB3 concentration. The color codes and n numbers are the same as in panel (B).

(D) Normalized averaged tip intensity profiles for His-GFP-MTLS along MTs at different times in presence or in absence of GUVs. The profiles were normalized to the maximum intensity at time corresponding to the first video acquired after the addition of the GUVs (t=0). In presence of GUVs t=0, n=17; t=10 min, n=25; t=20 min, n=18. In absence of GUVs EB3:50, t=0, n=28, t=30 min, n=39, t=45 min, n=23. EB3:50, t=0, n=15, t=15 min, n=51, t=30 min, n=33.

(E) Top: Snapshot of a TIRF microscopy time lapse video. Bottom: MT tip intensity profile (red dots) and the same profile after the maximum intensity was assigned to each of the preceding points along the MT (blue dots). The transformed profile was fitted with the error function (black line). Figure illustrates the fitting of an individual intensity profile by the error function.

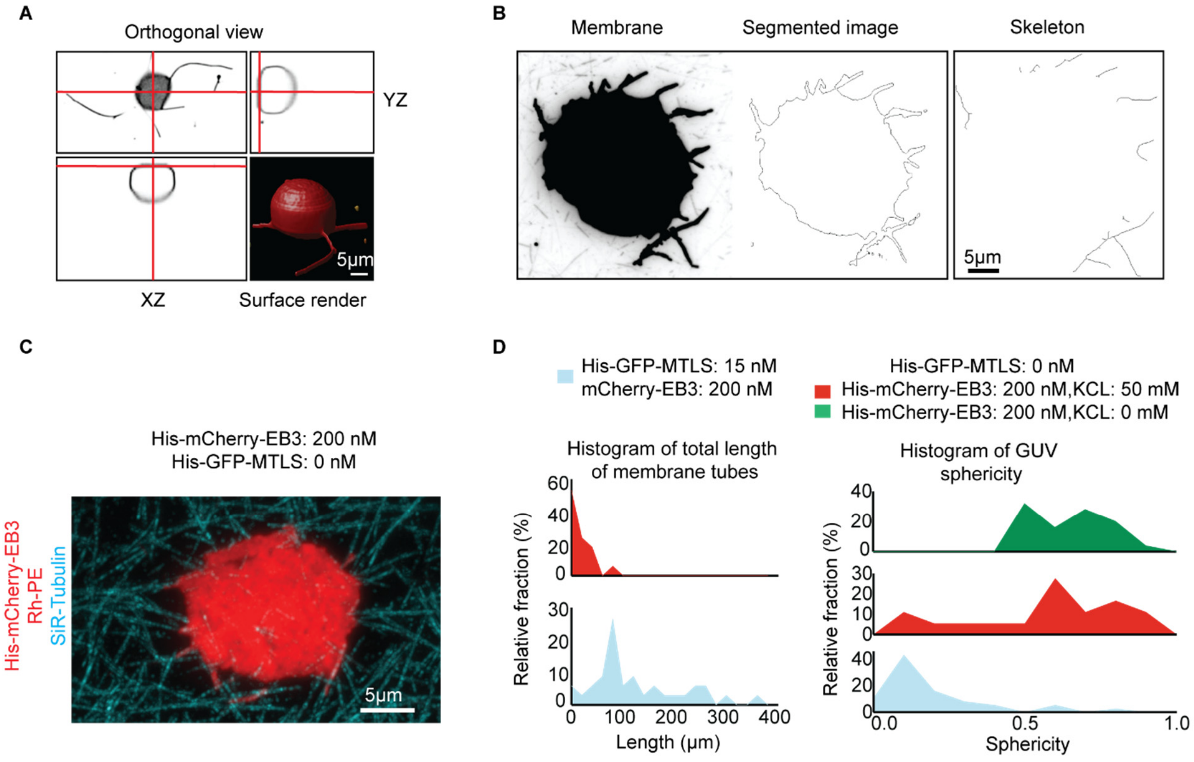

Figure S2. **Analysis of membrane tubulation, Related to Figure 1.**

(A) Orthogonal view and surface render of a confocal Z stack obtained using spinning disk microscope from a GUV after the incubation with dynamic MTs in presence of 200 nM mCherry-EB3 and 15 nM His-GFP-SxIP.

(B) Left: Snapshot of a tubular membrane network. The image is the maximum intensity projection of 100 frames of a time lapse video. Center: Segmented tubular network. Right: Skeletal representation of the tubular network.

(C) Still image of a GUV (red) in the presence of dynamic MTs (blue) and 200 nM His-EB3.

(D) Histograms of total length of membrane tubes (left) and GUV sphericity (right) measured at 200 nM His-EB3, in a buffer without added KCl (n=25 GUVs (green)), at 200 nM His-EB3 in the buffer supplemented with 50 mM KCl (n=34 GUVs (red)), and at 200 nM mCherry-EB3 and 15 nM His-GFP-MTLS, with 50 mM KCl (n=18 GUVs (blue)) .

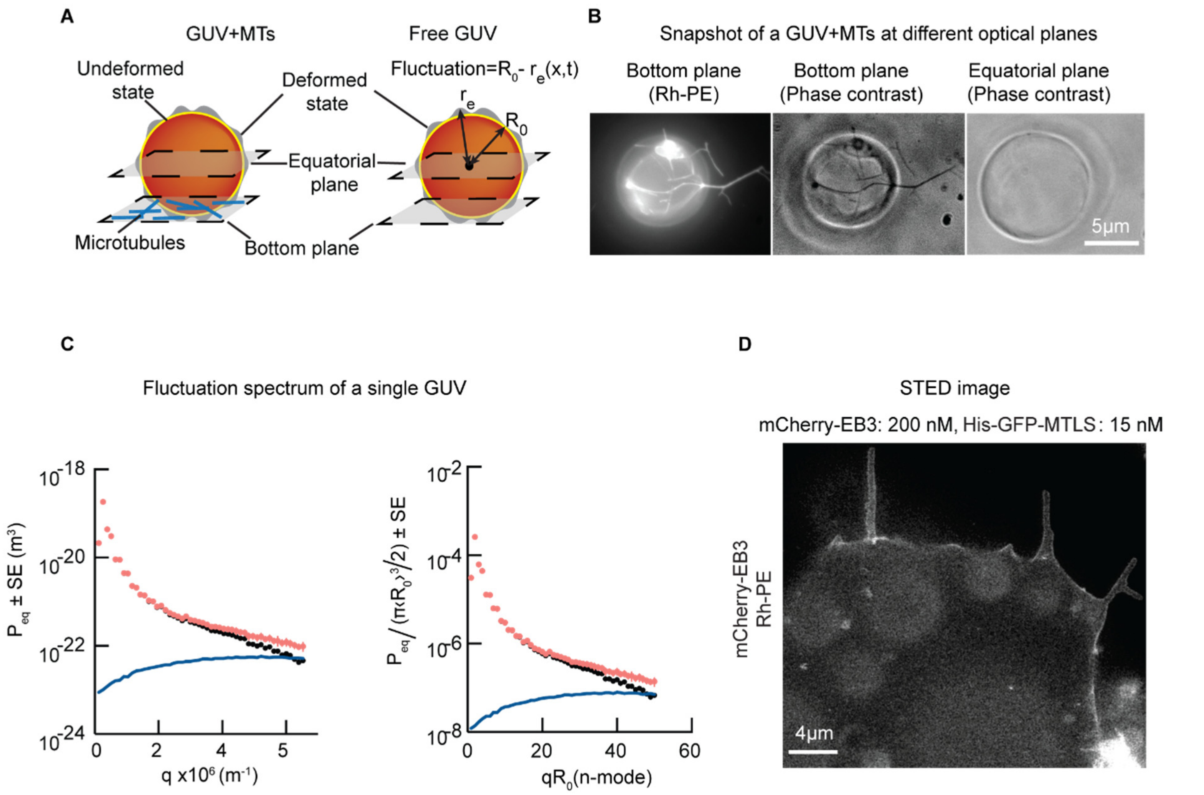

Figure S3. GUV fluctuation spectra and super-resolution imaging of membrane tubes, Related to Figure 2.

(A) Schematic representation of the two conditions where membrane fluctuations were investigated, GUVs in the presence of MTs and free GUVs. The equatorial and the bottom plane are indicated; blue lines represent MTs. The membrane fluctuates around an equilibrium position (undeformed state, yellow line) defined by the time-average of the GUV radius R_0. Fluctuations at each point (deformed state) are represented in grey and are defined by local deviations of the equatorial radius r_e from the average radius R_0.

(B) Phase contrast and fluorescence images of a GUV interacting with MTs. Bottom plane is shown on the left and center, equatorial plane is on the right.

(C) (Left) Experimental fluctuation spectrum calculated from the time-averages of the quadratic fluctuation amplitudes of the equatorial modes (red dots) measured for a single GUV with a radius of 10 µm. The blue line is the systematic contribution of the pixelization noise to the experimental fluctuation amplitude, which increases with the mode *n*. Black dots represent the amplitudes of fluctuations corrected for the pixelization noise. (Right) The spectrum was normalized by the factor $A=\pi\langle R_0\rangle^3/2$.

(D) Imaging of membrane tubes using STED microscopy. The images were acquired at 200 nM mCherry-EB3 and 15 nM His-GFP-MTLS.

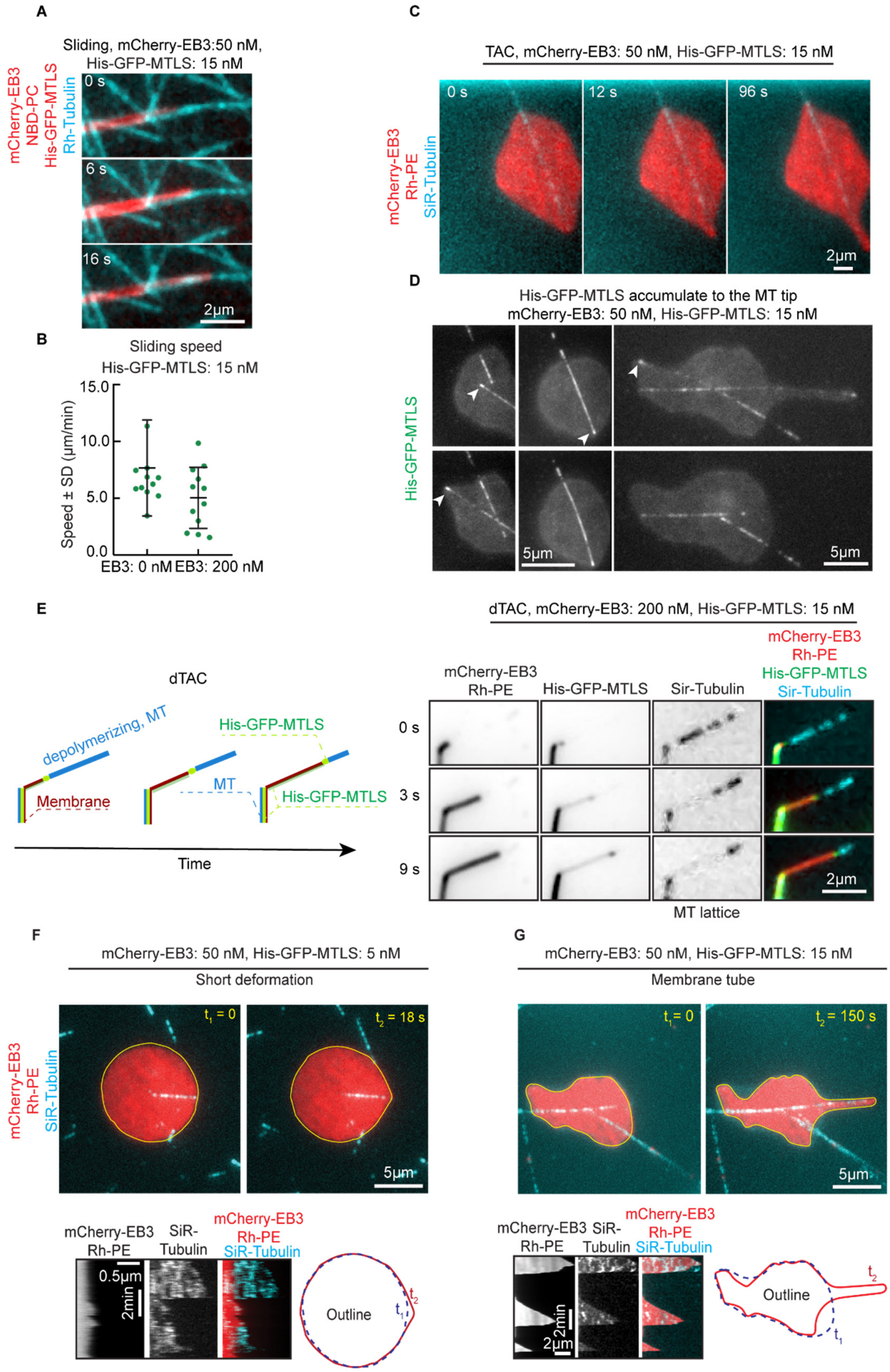

Figure S4. Three mechanisms of MT-induced membrane tube formation, Related to Figure 3.

(A,C,E) Time lapse images of a membrane tube moving along a MT shaft (sliding, A); together with the plus end of a growing MT (TAC, C) or by attachment to the plus end of a depolymerizing MT (dTAC, E). Schematic representation of the membrane tube and the MT is shown on the left in (E).
(B) Speed of membrane sliding at 0 nM (n=9), and 200 nM (n=12) EB3 and 15 nM His-GFP-MTLS.
(D) Snapshot of a GUVs in contact with the MTs. The white arrowhead shows the accumulation of the His-GFP-MTLS protein at the tip of the MTs. Only the His-GFP-MTLS channel is shown.
(F,G) Snapshots (top) and the corresponding kymographs (bottom) of a MT deforming a GUV at 50 nM mCherry-EB3 and 15 nM His-GFP-MTLS. GUV contours are shown by yellow lines. (F) The membrane detaches from the MT before developing a tubular shape. (G) The membrane remains attached to the MT tip during growth and shrinking phases. See also Video S4 and S6.

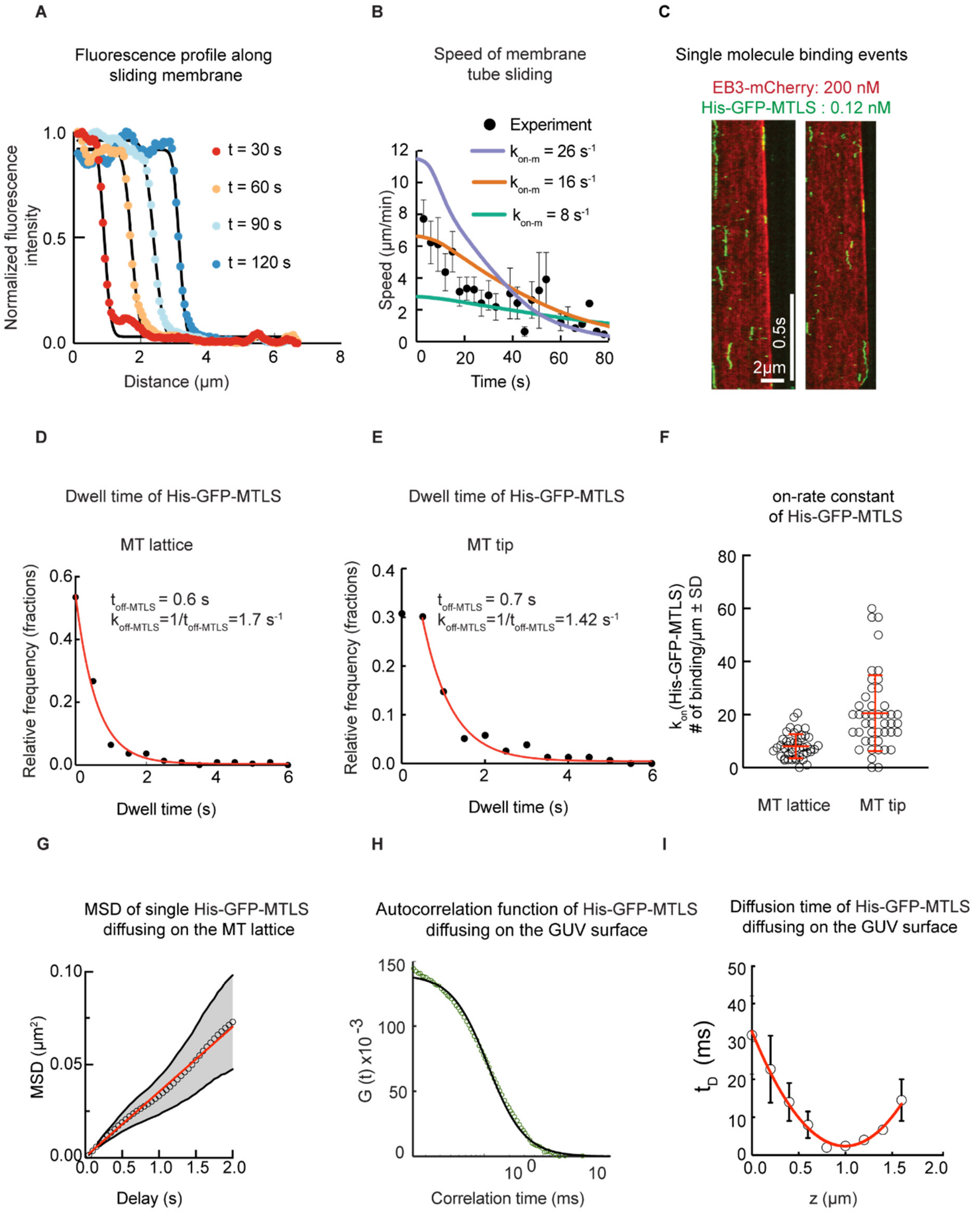

Figure S5. Determination of parameters used for modeling and stochastic simulations, Related to Figure 5.

(A) Membrane tip intensity profile at different time points measured from a GUV sliding along a MT shaft in presence of 15 nM His-GFP-MTLS without EB3. The position of the membrane moving along a MT shaft was determined from the fitting of the intensity profiles to the step function.
(B) Comparison between curves representing the speed of membrane tube sliding along MT lattice modeled using different association rates and the experimentally determined membrane sliding speeds. Experimental values of the average tube velocity are shown with black dots; error bars represent SEM, n=12.
(C) Kymographs of dynamic MTs grown in presence of 0.2 nM His-GFP-MTLS and 200 nM mCherry-EB3.
(D-E) Distributions of dwell time of single His-GFP-MTLS molecules at MT lattice (D) and growing MT plus ends (E); experimental conditions were the same as in panel (C). The red lines represent monoexponential fits.
(F) Association rate of the His-GFP-MTLS on MT lattice and growing MT plus ends, expressed in number of binding events per length.
(G) Mean square displacement (MSD) for single His-GFP-MTLS molecules diffusing on MT lattice. The red line represents a linear fit. Experimental values of the MSD are shown with open black dots. The shaded area represents SEM.
(H) Representative autocorrelation function obtained from the diffusion of the membrane-bound His-GFP-MTLS molecules (open green dots). The black line represents a fit to Equation 3 (see STAR Methods).
(I) Diffusion time of the membrane-bound His-GFP-MTLS molecules measured by FCS as a function of the position in z. Experimental values of the diffusion time are shown with black open dots. The red line represents fit to Equation 4 (see STAR Methods).

Parameter		Description	Value	Source
1	d	Length of the tubulin dimer	8 nm	Model assumption, see Methods
2	k_{off}	Dissociation rate of the MTLS from the MT lattice	1.7 ± 0.1 s^{-1} (n=217)*	Measured experimentally from single binding events Figure S5D
3	k_{off-m}	Dissociation rate of the MTLS bound to GUV from the MT lattice	$k_{off-m} = k_{off}$	Measured experimentally from single binding events Figure S5D
4	$k_{on-m} = (\max(V_{spreading})/(d)) + k_{off-m}$	Association rate of the MTLS with the MT lattice	16 ± 8 s^{-1} (n=12)*	Measured experimentally. Figure S5A,B
5	$k_{on-m-tip}$	Association rate of the MTLS with the MT tip	40 s^{-1}	Based on experimental data see Methods
6	ξ	Characteristic length of the potential barrier	1 nm	Model assumption, see Methods
7	n_p	Number of MTLS molecules bound at the tip	1-3	Model assumption, see Methods
8	σ	Lateral membrane tension	[2e-7,2e-6] N/m	Based on experimental data, Figure 2F
9	κ	Bending rigidity of the membrane	[4e-20, 3e-19] J	Based on experimental data, Figure 2F
10	R	GUV radius	5-15 μm	Based on experimental data
11	r_0	Curvature radius of the tether	Calculated as $r_0=(\kappa/2\sigma)^{1/2}$; Ref. [S1]	Based on experimental data
12	p_{m-MTLS}	Occupation probability of MTLS along a MT starting from the MT tip	Experimentally measured function of distance from microtubule tip	Based on experimental data, see Methods, Figure 6B,C
13	$<N_{p-tip}>$	Maximum number of MTLS molecules at the MT tip	28 ± 18 (n=19)**	Based on experimental data
14	D_{MT}	Diffusion coefficient of MTLS molecules along the MT	$0.03 \pm 0.01 \mu m^2/s$ (n=104)*	Measured experimentally from single binding events Figure S5G
15	k_{D-MT}	Diffusion rate of MTLS molecules along the MT	468 ± 156 s^{-1}*	Based on experimental data
16	D_m	Diffusion coefficient of MTLS molecules bound to the membrane	4 ± 2 $\mu m^2/s$ (n=18)**	Measured experimentally by FCS Figure S5H,I
17	k_{D-m}	Diffusion rate of MTLS molecules bound to the membrane	56 ± 31 ms^{-1} *	Based on experimental data
18	w_0	Radius of the laser beam in the focal plane	0.20 ± 0.06 μm (n=18) **	Measured experimentally by FCS Figure S5H,I
19	N_{MTLS-m}	Number of MTLS molecules bound to the GUV surface measured at 15 nM MTLS (measured inside of the focal volume)	5 ± 5 (n=18)**	Measured experimentally Figure S5H
20	$\rho_{MTLS} = N_{MTLS-m}/w_0$	Surface density of MTLS molecules bound to the GUV surface	125 ± 26 #molecules/μm^2 (n = 18)*	Based on experimental data

21	$\rho_{MTLS-MT} = \rho_{MTLS} * (I_{GUV-MTs\ contact} / I_{GUVS})$	Surface density of MTLS molecules bound to the GUV surface in contact with MTs	250 ± 112 #MTLS/μm^{2}*	Based on experimental data Figure 2B

Table S1. Parameters of the model and simulations, Related to Figure 5 and Figure 6.

The table contains the parameter values used in the analytic model and simulations. Dimeric His-GFP-MTLS protein (abbreviated as MTLS in the Table) was used for all measurements.
*mean value ±SE, **mean value ± SD

Supplemental References

S1. Derényi, I., Jülicher, F., and Prost, J. (2002). Formation and interaction of membrane tubes. Phys Rev Lett *88*, 238101.
